# Dictionary of Practical Medicine

**Published:** 1847-04-01

**Authors:** 


I, Dictionary of Practical Medicine. By *James Copland*, M.D., F.R.S., &c. &c. Parts X. & XI. Article, Pestilence. London, Longman & Co. 1846-7.

II. A Treatise on the Plague; more especially on the Police Management of that Disease, illustrated by the Plan of Operations successfully carried into Effect in the late Plague of Corfu. With Hints on Quarantine. By *A. White*, M.D., Deputy Inspector-General of Hospitals, &c. Octavo, pp. 342. London, Churchill, 1846.

There is no subject in the whole range of Medical Science of such wide and momentous interest, alike to the physician and to the public at large, as the history of Pestilential Diseases—including their probable mode of origin; the various circumstances of climate, season, locality, &c., which seem either to promote or to check their development; the causes of the changes or phases which they occasionally exhibit; the laws of their propagation and diffusion; their influence upon population; the effect of quarantine and other restrictive measures upon their progress; the operation of medicinal and hygienic treatment upon individual cases, and also upon the general mortality in a community; as well as various other questions of minor note that must suggest themselves in so comprehensive an enquiry. By the term "Pestilence," has generally been understood any malignant and rapidly fatal *Epidemic*, or extensively diffused distemper. It has been used, therefore, not so much to designate any particular disease or set of diseases, as to denote the general feature or character of their prevailing within a short period over a large extent of space, and of their proving, at the same time, highly destructive. Dr. Copland however employs the word in a more limited, and in a somewhat peculiar, sense. "Under the head *pestilence*," says he, "I comprise certain maladies which have appeared as wide-spreading and devastating epidemics, but which have surpassed all other epidemics in their rapid extension, in their fatality, and in the duration of their prevalence." He goes on to remark that these "certain maladies," although usually prevailing epidemically, may occur also in a sporadic form. The attribute of *epidemicity* might thus seem to be not necessary to the existence of a pestilence; but Dr. C. guards himself against this objection, by stating that it is to "the irruptions of these maladies, to their rapid extension, and to their great fatality, the generic term *pestilence* may be justly applied." Why Dr. C. should have deviated from the ordinary view of the question, and also have limited the term to three diseases—viz. Epidemic Cholera, Yellow Fever, and the Plague, or, as he calls them, the Choleric Pestilence, the Hæmagastric Pestilence, and the Septic or Glandular Pestilence—is not very apparent; unless, indeed, he considers that this triad is possessed of certain characteristic features in common, peculiar to themselves, and distinguishing them from all other maladies to which the appellation of "pestilential" has usually been applied. Whether he has acted wisely in so doing, we shall not now wait to enquire. That this arrangement has had its effect in leading Dr. Copland to adopt some very peculiar views, as to the origin and diffusion of the diseases in question, will appear when we come to examine the details which he has adduced upon these very important subjects. He loses no time, we may remark, in announcing, at the very threshold of the enquiry, his strong and decided opinion, not only that the three pestilences named are essentially *infectious*, but also that this property of *infectiousness* has always operated as the most influential cause in their dissemination. The reader is thus at once prepared for what awaits him in the perusal of the sequel: we need scarcely say that our author displays his accustomed industry and erudition in endeavouring to substantiate the views which he has adopted. It is to a patient and candid examination of the proofs and arguments which he adduces, that we now invite the attention of our professional brethren.

As it is most necessary, in scientific discussions, that the exact signification of the technical terms or phrases employed should be clearly understood by all parties, we have to observe that, whenever the words *infectious, infection,* &c. are used, we wish to denote that property of certain diseases being communicable from one individual to another, in consequence of certain effluvia or morbific miasms emanating from the body of the sick, and acting, through the medium of the respired air, upon that of a person in health. Scarlatina, Measles, and Hooping-cough, are universally admitted to be *infectious* diseases, in this acceptation of the word. The epithet *contagious,* on the other hand, ought assuredly to be restricted to such diseases, as are communicable only by *contact,* and not by any vitiation of the atmosphere with morbific effluvia, in the manner we have just alluded to. We would suggest the term *contagio-infectious,* to denote those that are capable of being communicated in both ways. To this latter class belong Small-pox, Hospital Gangrene, and perhaps also Glanders and Puerperal Fever. The importance of attending to these distinctions will be perceived in the course of our enquiries, more especially when we come to notice (once more) the last of the three Pestilences on Dr. Copland's list.

But, before concluding these prefatory remarks, we must not fail to allude to a circumstance of the very highest interest in the general history of Infection, and one which requires to be most assiduously kept in mind whenever an attempt is made to determine whether this property belongs to certain diseases or not. The point, to which we refer, is thus very accurately set forth by Dr. Copland in the following passages, from the article "Infection," in his elaborate work:—

"It may be stated as an axiom that the foul air, generated by the crowding of many persons or animals even in health, but more especially in disease, into a confined space, as in the wards of hospitals, &c., or by few persons only in the same apartment, if their diseases be attended by copious discharges, will infect those who breathe it in a state of predisposition, with low fever, dysentery, &c.; and that the persons thus infected will communicate the malady to others similarly predisposed."

Again, we thus read in another passage:

"Diseases may take place sporadically, or from local causes; and, owing to various circumstances acting either in close succession or coetaneously, the circulating and secreted fluids, and even the soft solids, may be so changed during its course as to emit an effluvium contaminating the surrounding air, and thereby infecting many of those who breathe this air in a sufficiently contaminated state; and thus it will be propagated to several, and from these to others—especially under favourable circumstances of temperature, humidity, electrical conditions, and stillness of the air, and of predisposition on the part of those who come within the focus of infection. Thus diseases *may become infectious and epidemic*, aided by the constitution of the air and other circumstances; and, after a time, cease and entirely disappear with the circumstances which combined to propagate it."

This very lucid description of what is usually meant by the expression "contingent" or "conditional infection" deserves especial notice, as bearing, in no indirect manner, on the history of Epidemic Diseases in general.* Other passages, inculcating the same views, might be quoted from the Dictionary of Practical Medicine; but these now given will amply suffice.

---

* In a recently-published pamphlet we expressed the same doctrine in the following terms:—

"Whenever a number of human beings, even in a state of health, are cooped together in a narrow, ill-ventilated space, the air gradually becomes so contaminated by the effluvia given off from their bodies, that, in the course of a more or less limited space of time, Fever will almost inevitably make its appearance among them; and this fever, so generated, will often be found to exhibit *infectious* properties, if the sick are not removed to a more airy and wholesome locality. Something of this sort was observed in the case of most of the 23 survivors of that dreadful night, when upwards of 140 human beings were shut up in the Black Hole at Calcutta. We have daily illustrations of the same fact in what takes place on board troop and slave ships, in jails, crowded penitentiaries, and so forth.

"If such then be the case with persons in health, can we wonder that the effluvia from the bodies of the sick must be still more poisonous and contaminating? If any one has a doubt upon this score, let him walk from the open air into the ward of an hospital, when all the windows have been closed for a time; a sense of nausea and oppression, accompanied not unfrequently with actual shivering, are often immediately experienced.

"Now, it is in the way we have just mentioned that various maladies, which are certainly not primarily or essentially infectious, are apt to become so in impure and badly-ventilated situations, more especially when many sick are crowded together. The infectiousness is not a necessary quality of the disease; it is an

Now, if the doctrine of Contingent Infection be admitted—and where is the enlightened reader who will dispute its truth?—it is obvious that, to talk of medical men being of a necessity either "infectionists" or "non-infectionists," in reference to certain diseases—as if they must positively make up their minds either to admit or to reject *in toto* the communicability of these diseases under all circumstances—is not dealing quite fairly with the question. To call those who hold the doctrine just mentioned "non-infectionists," is clearly to mislead the unpractised reader, by leaving an erroneous impression upon his mind. Let us take an example. If the broad and general question, "Is Erysipelas or is Dysentery infectious"? were put to a physician, he might with perfect truth and propriety answer it in the affirmative; and yet all that he meant by such a response might be the simple declaration of his opinion that these diseases are occasionally or conditionally communicable from one person to another, although such an occurrence does not happen once in a thousand times, and only under very peculiar circumstances. It would be wrong, however, to assert, without any qualification, that the physician in question was either an infectionist or a non-infectionist. Now, as it is with Erysipelas and Dysentery, so it may be with Malignant cholera. To adduce a few instances wherein there has seemed to be a transmission of the disease from one individual to another—and this, too, be it remembered, not in *sporadic* cases, but when an acknowledged *epidemic* is prevailing at the time—cannot satisfy any dispassionate enquirer that the disorder should be placed in the same category with Scarlatina or Measles. But the mere determination of the question, whether a disease like Pestilential Cholera is *ever* infectious, is far from being either the most difficult or most important point in our enquiries. We have to ascertain whether this property of infectiousness is primary, essential, and permanent, or only contingent, conditional, and occasional;—whether the attacks of the pestilence can, with any shew of probability, be attributed to personal infection from one individual to another;—whether its dissemination from one district to another, and from one country or continent to another, can be fairly traced to this agency;—and whether there be any rational grounds for hope that its progress may be arrested, or its invasion prevented, by any of those means of restraint or precautionary regulations, comprised under the general term of Quarantine. These are the really momentous problems in the enquiry to be discussed; and not the very minor one, whether the disease has, upon any occasion, manifested infectious properties. Medical men have too often allowed themselves to be altogether occupied with the history of insulated facts, and have neglected to view the question in its

---

accessory or contingent attribute. Various forms of low or typhoid Fever, Erysipelas, Dysentery, Angina, &c. may be mentioned as affording not unfrequent examples of the phenomenon in question. The consideration of this subject teaches us two important lessons. In the first place, it inculcates the imperious necessity of thorough cleanliness and free ventilation, wherever a multitude of sick persons are congregated together; and in the second, it exposes the absurdity of the disputes which have so often taken place about the infectiousness or non-infectiousness of several diseases."—*Quarantine and the Plague,* 8vo. pp. 71. Highley, 1846.

broad and more general bearings. They seem to have been examining the eddies by the shore, and to have forgot all the while to watch the great current of the ocean stream.

With the view of keeping our reader's attention steadily directed to the topic most worthy of consideration, we shall occasionally in the sequel employ the terms "importationist" and "non-importationist," in place of those of "infectionist" and "non-infectionist," in more common use.

Having cleared our way of any difficulties arising from the ambiguity of certain technical expressions, we should now at once proceed to examine the arguments adduced by Dr. Copland to shew that Pestilential Cholera is not only essentially and primarily communicable from one person to another, but also that its wide diffusion and extension have been chiefly attributable to this property of infection. But, before we can do this with any advantage, we must first investigate a point in the history of the disease on which our author dwells with great emphasis, and the right determination of which is very intimately connected with our ulterior enquiries.

Dr. Copland divides Cholera into three species; 1, the bilious; 2, the flatulent; and 3, the spasmodic, or *mort de chien* of East Indian writers; while what has been generally termed the Epidemic or Pestilential disease is declared by him to be not Cholera at all, but a distemper that is *sui generis*, essentially and fundamentally distinct not only from the various species of Cholera now enumerated, but also from all other diseases, and which he proposes to designate by the appellation of "Asphyxia pestilenta." He employs indeed the term of "Choleric Pestilence; but only for the sake of convenience. Now the main question comes to be, how are we to distinguish between the malignant or aggravated form of endemic Asiatic Cholera, so well known to, and so well described by, many E. India writers during the latter part of last century and the beginning of the present one, and this Pestilential disease, which Dr. Copland considers to be of very recent origin and of an essentially different nature. Let us see what he says upon the subject; and, first of all, we shall look at the definitions which he has given of these two fundamentally-distinct disorders.

"Spasmodic Cholera" is defined thus: *vomiting and purging of watery matters, without any appearance of bile; spasms violent and extending generally through the frame;* (it is subsequently said, "spasms of a violent, painful and tonic character, attacking the muscles of the abdomen, thighs, legs, thorax, and lastly the arms and hands"); *speedily followed by sinking of the powers of life.* This disease is *endemic* in some inter-tropical countries. Sometimes, Dr. C. says, it has prevailed *epidemically;* and then he admits that it "nearly approaches" the pestilential disease; viz. the Asphyxia pestilenta. *He does not consider it to be infectious.* Let the reader particularly mark this point.

On the other hand, the definition of the "Pestilential Disease" stands thus:—"*Anxiety and oppression in the chest, epigastrium and præcordia; disturbance of the bowels, with nausea, faintness, giddiness, and depression of vital power; frequent ejections of an offensive fluid, resembling rice-water, from the bowels and stomach, followed by spasms, tremors, distress; a cold, clammy, purplish, and shrivelled state of the surface; coldness and rawness of the expired air; a sense of painful or burning heat at the epigastrium, with*

*urgent thirst, and rapid disappearance of the pulse;—the distemper being often preceded by indigestion and diarrhœa, and frequently followed by febrile reaction, affecting chiefly the brain and abdominal organs.*"

The one definition is certainly considerably fuller than the other; but where, it will be asked by many, is the material difference between them? In the latter, the general appearance of the patient, the state of the surface of the body, and the rapid sinking of the pulse are made prominent characteristics; but then, if we turn to Dr. Copland's own description of the bad cases of the Spasmodic Cholera, what do we read?

"In the course of a few hours," says he, "the features shrink, the hands and feet become cold and clammy, the exacerbation of the spasms force out a cold clammy sweat on the face and breast, the pulse is extremely small and weak, or nearly disappears;—in a case which came before me in Africa in 1816, the pulse could scarcely be felt four hours from the attack. * * * * The powers of life fail very rapidly; the eyes sink and are surrounded with a livid circle; the countenance assumes a remarkably anxious cast, or is pale, wan, and shrunk; and the spasms extend to the very fingers. The breathing now becomes extremely laborious; the patient is restless, and at last is carried off, sometimes in the space of ten or twelve hours."

The accuracy of Dr. Copland's description is well shewn by comparing it with the accounts left us by Curtis, Girdleston, Duffin, and other medical men who wrote upwards of seventy years ago. Take that of Curtis, a naval surgeon, writing of the *mort de chien*, or the malignant endemic cholera, at Trincomalee in 1782:

"In all of the cases, the disease began with a watery purging, attended with some tenesmus, but with little or no griping. This always came on some time in the night, or early towards morning, and continued some hours before any spasms were felt. * * * * * * * The purging soon brought on great weakness, coldness of the extremities, and a remarkable paleness, sinking and lividity of the whole countenance. Some at this period had some nausea and retching to vomit, but brought up nothing bilious. In a short time, the spasms began to affect the muscles of the thighs, abdomen and thorax, and lastly they passed to those of the arms, hands and fingers. * * * The hands now began to put on a striking and peculiar appearance. The nails of the fingers became livid and bent inwards; *the skin of the palms became white, blanched, and wrinkled up into folds, as if long soaked in cold water.* * * * * * All this while, the purging continued frequent, and exhibited nothing but a thin watery matter or mucus. In many, the stomach became at last so irritable that nothing could be got to rest upon it; but every thing that was drank was spouted out immediately, without straining or retching. The countenance and extremities became livid; the pulsations of the heart more quick, frequent and feeble; the breathing began to become more laborious and panting; and, in fine, the whole powers of life fell under such a great and speedy collapse, as to be soon beyond the power of recovery."

Girdleston has described with equal accuracy the disease, as observed by him in the naval hospital at Madras, in the course of the same year, 1782. One passage in his description is remarkable.

"The hands and feet generally become sodden with cold sweat; the nails livid, the pulse more feeble and frequent, *and the breath so condensed as to be both seen and felt, issuing in a cold stream at a considerable distance.* The thirst was insatiable, the tongue whitish but never dry; vomitings became almost incessant, the spasms, cold sweats and thirst increased with the vomitings. * * * * * * Some died in the first hour of the attack; others lived a day or two with remissions."

It would be easy to adduce from other writers descriptions of the *mort de chien,* corresponding in almost every particular with the definition of the Pestilential disease given by Dr. Copland. But this must surely be unnecessary after the preceding details. We shall therefore only allude to the published evidence of one or two gentlemen who were in India for several years before, as well as after, the outbreak of the great epidemic of 1817—at which period our author supposes that his "Asphyxia pestilenta" first manifested itself,—and who must therefore have been fully competent to judge of the question under consideration.

"The disease in the 9th Reg. N. I. in 1814," says Mr. Duncan, in a report, dated September 1819, "resembled in every particular (with the exception of the heat at the præcordia) the Cholera at present so common, although it could not be called epidemic."

Mr. Cruickshank has given a more minute description of the disease of 1814. The following is an extract from a report that was sent in by this gentleman to the Madras Government.

"When taken into hospital," Mr. C. observes of the first cases he saw, "they exhibited all those symptoms, now so well known, of persons labouring under the advanced and fatal stage of Epidemic Cholera; the skin cold, and covered with cold perspirations; the extremities shrivelled, cold, and damp; the eyes sunk, fixed, and glassy, and the pulse not to be felt. These persons all died, and I find, on referring to such notes as I have preserved, that, influenced by consideration of the vascular collapse and total absence of arterial pulsation, I had denominated the disease Asphyxia. Many sepoys were brought into hospital in circumstances approaching to those above detailed. Of them, in a considerable proportion, the disease terminated fatally. Thus the cases which I first saw of this malady, in the aged among the camp-followers, differed in no respect from the worst cases of that affection since so well known under the name of Spasmodic Cholera. That name, however, I did not adopt, neither in my public reports nor in the private notes which I took at the time. In this I was chiefly influenced from considering the nature of the matter ejected by vomiting and by stool, which in cholera is said to consist of bile, but which in these cases was aqueous or mucilaginous. Besides, it was evident that the diluent treatment, recommended in cholera, would never be applicable to such a disease as that with which I had to contend. I continued, therefore, to employ in my reports the term 'bowel-complaint,' both because I found it in the hospital-books on joining the corps, and because, if it conveyed no very precise idea of the malady which it was meant to designate, it was at least an appellation whence no erroneous impressions could be derived."

After the perusal of these extracts—and they might easily be multiplied, if need were—few readers, we should suppose, will be able to discover for themselves the diagnostic differences of two diseases, which are, however, declared by our learned author to be essentially distinct from each other. That he should, notwithstanding such evidence (with which he must be perfectly well acquainted), heroically maintain that there is "a marked distinction" between the malignant form of endemic Cholera, known by the name of the *mort de chien,* and the epidemic disease which has attracted so much notice for the last 30 years, seems indeed very surprising. Yet, he confidently assures us that he is fully convinced of the fact. Nay, he does not hesitate boldly to declare that in the correctness of his opinion—viz. that the former disease does not exhibit the pathognomonic symptoms of the latter—" he is borne out by the experience of every well-informed and candid observer, who has seen the disease in this country." Strange assertion !—from one, too, who has never witnessed a single instance of Spasmodic Cholera in the East Indies, and in the very teeth of such men as Dr. Johnson and Mr. Ranald Martin—not to enumerate a host of other Oriental authorities—who have expressly declared the very contrary in their writings. Indeed, we in vain search for any testimonies among our East Indian practitioners in support of this singular dogma of Dr. Copland. On this ground alone, therefore, we might very fairly declare it to be utterly erroneous. But, as the correct solution of the point at issue has a very direct bearing upon the still more important question of the alleged infection (the *importable* infection, be it always remembered) of the disease, we are disposed to devote a page or two more to the subject, with the view of giving an ample opportunity to our author to produce his reasons for the singular opinion which he has adopted. In the section on the Diagnosis of the Pestilential Cholera, we come to the following passage :—

" In the Spasmodic or severe form of sporadic* Cholera, the discharges from the stomach and bowels are certainly either not coloured by bile, or but little, excepting at the commencement, and when the disease begins to yield ; but they are accompanied with a different train of symptoms. The spasms are more tonic, and confined more to the muscles of the abdomen and of the thighs and legs, than in the Pestilential disease : and, in the former, the vertigo, deafness, headache, marked affection of the respiratory function, and of the circulation, characterising the latter, are entirely wanting.

" In sporadic or Bilious Cholera, the very dark, thick, and ropy appearance of the blood ; the cold, wet, and shrivelled state of the surface, and its leaden, dark, or purplish colour ; the almost total absence of pulse at the wrist ; the very marked and rapidly increasing collapse of the powers of life ; the disagreeable and earthy colour of the body, even during the life of the patient ; the burning sensation between the scrobiculus cordis and umbilicus ; the complete arrest of the glandular secretions ; the cold tongue and mouth ; and the coldness of the respired air, which characterise the Pestilential disease, are entirely absent."

It is scarcely necessary, we should think, to offer any comments upon these remarks. No one requires to be told how to distinguish the pestilential from the common bilious form of Cholera : the difficulty lies in distinguishing the former from the spasmodic or malignant form of the endemic disease of the East Indies. We doubt much whether " the vertigo,

---

* The epithet " sporadic" cannot be correctly applied, at least by Dr. Copland, as a distinctive appellation of the malignant endemic Cholera ; for he has already admitted, on the one hand, that this disease occasionally prevails *epidemically*, and, on the other, that the Pestilential disease sometimes appears *sporadically*. It would almost seem from this misapplication of the term by our learned author that, in spite of his strong prepossessions to the contrary, he had unconsciously been led to recognise the truth of the general opinion, that Malignant Cholera and his new Pestilence are one and the same disease ; the former being its sporadic, the latter its epidemic form.

deafness, headache, marked affection of the respiratory function and of the circulation," that are alleged to be characteristic of the former, will much assist the practitioner at the bed-side of his patient. As to the gratuitous assumption that "the absence of the bile" in the one disease "is to be imputed to spasm of the common biliary duct rather than to a suppression of the secreting and excreting functions, whilst in Pestilential Cholera, these functions are altogether arrested," it is quite obvious that it will never do to rest a diagnostic discrimination upon any hypothetical distinctions. We must look at facts, not fictions, in forming an opinion upon such a point. Now, what says our author himself—not to quote any foreign or extrinsic authority—upon the necroscopic state of the biliary organs in these two diseases, that are said to be "different in all their relations"? In the description of Spasmodic Cholera, given at page 322 of the 1st vol. of the Dictionary, we are told that, "in fatal cases, the liver has been found congested, the gall-bladder and hepatic ducts filled with dark-coloured inspissated bile;" and at page 106 of the 3rd volume, in the account of the *post-mortem* appearances of the Pestilential Disease, we read that "the liver is generally pretty full of dark-coloured blood; the gall-bladder often much distended with tenacious ropy bile, of a dark yellow or green colour." Where, pray, is the difference between these two statements? Dr. C. adds, "the urinary bladder is always contracted and empty." The same necroscopic appearance was observed in fatal cases of malignant Cholera 70 years ago; for we are expressly informed by Mr. Davis, who a was a member of the Madras Hospital Board in 1787, that "the bladder was most singularly contracted, and did not exceed in size a large nutmeg."

There is another alleged diagnostic mark upon which Dr. C. lays much stress. He says: "The *secondary fever and consecutive phenomena,* which follow upon the cold and blue stage of the malady, also furnish remarkable proofs of dissimilarity between the Pestilence and the severe forms of Cholera observed in hot countries........ After these latter, the patient recovers without any consecutive disease, and frequently the tumult of the frame leaves it benefited by the changes it induces; but, in the present pestilence, the consecutive states of the disease are as dangerous as the blue stage." Now, Dr. Copland does not require to be informed that the secondary fever, to which he alludes, was of infinitely greater frequency in Europe than in India; and, indeed, this very circumstance has been particularly dwelt upon by almost all writers on the subject, as one of the most prominent features of difference between the disease as seen (more especially on its first outbreak) in its primary and indigenous seat in the East, and when it became transplanted into more temperate climates; it appeared to assume something of the type of the endemic fevers of the countries which it visited. Without pursuing this subject, we shall merely give one short passage from the Madras Report in confirmation of the first clause of this sentence.

"When medical aid is early administered, and when the constitution is otherwise healthy, the recovery from an attack of (epidemic) Cholera is so wonderfully rapid, as perhaps to be decisive of the disease being essentially unconnected with any organic lesion. In natives of this country especially, in whom there is ordinarily very little tendency to inflammatory action, the recovery from Cholera is generally so speedy and perfect that it can only be compared to recovery from syncope, colic, and diseases of a similar nature; but in Europeans, in whom there is a much greater tendency to inflammation and to determinations to some of the viscera, the recovery from Cholera is by no means so sudden or so perfect."

We have already seen that Dr. C. admits that the Spasmodic Cholera or *mort de chien* sometimes prevails *epidemically*, and that then it appruaches very nearly to the pestilential disease of 1817. As the admission of this circumstance is of very material consequence in investigating the true nature of the latter, it may be worth while to adduce one or two authentic instances of the fact mentioned, in the way of illustration. In the Bengal Report, we are informed that—

"A division of Bengal troops, consisting of about 5000 men, was proceeding, under the command of Colonel Pearse of the Artillery, in the Spring of 1781, to join Sir Eyre Coote's army on the coast. It would appear, that a disease resembling cholera had been prevalent in that part of the country (the Northern Circars), some time before their arrival; and that they got it at Ganjam on the 22nd March. It assailed them with almost inconceivable fury. Men, previously in perfect health, dropt down by dozens; and those even less severely affected were generally dead or past recovery within less than an hour. The spasms of the extremities and trunk were dreadful; and distressing vomiting and purging were present in all. Besides those who died, above five hundred were admitted into hospital on that day. On the two following days, the disease continued unabated, and more than one half of the army was now ill. In a note it is added, 'the occurrence of the disease on this occasion is noticed in a letter dated 27th April, 1781, from the Supreme Government to the Court of Directors; and the destruction which it caused in this detachment mentioned in terms of becoming regret.'

"After adverting to its progress in the Circars, the letter thus proceeds: 'The disease, to which we allude, has not been confined to the country near Ganjam. It afterwards found its way to this place (Calcutta): and after chiefly affecting the native inhabitants, so as to occasion a great mortality during the period of a fortnight, it is now generally abated, and pursuing its course to the northward.' It would have been interesting to have traced this disease, as it seemed to have put on the Epidemical form, but every attempt to discover its further progress has proved fruitless."

In the same Report it is stated, that, "in the month of April 1783, Cholera destroyed above 20,000 people assembled on occasion of a festival at Hurdwar." Sonnerat, in his travels, alludes to an epidemic which very closely resembled the recent pestilence, and which, in one visitation, carried off above 60,000 persons from Cherigam to Pondicherry! "The Indian physicians," he says, "could not save a single person." Mention is made by this author of a still more destructive outbreak of the pestilence, two years later. Whoever reads his narrative cannot hesitate for a moment in recognizing all the characters of Epidemic Cholera.

After quoting several other instances of a similar nature, Mr. Ranald Martin very emphatically remarks:

"It thus appears clearly that Epidemic Cholera prevailed *at various remote periods*, and at many of the principal stations throughout British India, sometimes coming as a wide-spread pestilence, and, at others, desolating only particular localities."* Yet in spite of all this, Dr. Copland does not hesitate to declare " that the accounts, which we possess of the epidemics and pestilences which have ravaged various countries (Hindostan, among the number; *Rev.*) in former times, do not furnish us with the history of any disease which may be considered as identical in its nature with this pestilence; and that it must, owing to this circumstance, and to the uniformity of its characteristic phenomena, be viewed as being of modern origin (1817), and *sui generis.*" Now, we are not surprised that Dr. C. so resolutely maintains the position which he has taken np; as it is quite obvious that, having admitted (as he has done) that the Spasmodic Cholera of the East is *not infectious*, he would be obliged, by the very force of common consistency, to view the Pestilential disease in the same light, if there be in truth no essential difference between the two maladies. We are quite willing to leave the facts already quoted, and the inferences plainly deducible from them, to speak for themselves.

Before, however, quitting this part of our subject, it may not be amiss to remind our readers that a malignant form of Cholera, resembling in most respects the endemic disease of the East, was not uncommon in this country in the time of Sydenham, who has described it with his usual accuracy. After relating its symptoms, and mentioning that it was almost invariably limited to the month of August, he very pointedly alludes to the marked difference between it and the more common bilious form of cholera, such as is met with at other times of the year; " as if," says he, " there lay concealed some peculiar condition of the air of this particular month (August), which is capable of communicating either to the blood or the ferment of the stomach a sort of specific alteration, adapted only to this disease." It is worthy of notice that the Bombay Report alludes, in very emphatic terms, to the resemblance in the features of the disease depicted by our English Hippocrates, and those of the epidemic from which India was at that time (1819) suffering; and Mr. Martin likewise has taken an opportunity of remarking that " many of the cases described by Sydenham would seem to have been of the true spasmodic nature."

The pernicious Intermittent Fevers also, so faithfully pourtrayed by several of the older physicians, occasionally exhibited a train of symptoms that bore a very close resemblance to those of malignant Cholera. It is impossible to read their descriptions without being struck with this resemblance, and admitting that the idea of Dr. Negri—" that the malignant cholera of our days belongs to the same class of diseases which was seen by Mercatus in Spain, Torti in Italy, and Morton in England"—is not entirely fanciful. But we have not to go so far back to find records of cases, exhibiting most, if not all, the features of Spasmodic Cholera. Without making any extracts, we may allude to the description given by Frank at the beginning of the present century, of what he calls " intermitting Choleric Fever;" also to the account of an Epidemic Cholera at Leeds in 1825, by Messrs. Thackrah and Dobson, in the Number of the

---

* The Influence of Tropical Climates, &c. by James Johnson, M.D. and James Ranald Martin, Esq. 6th Edition, p. 306, 1841.

Medico-Chirurgical Review for April 1832. We may likewise refer to the work of Dr. Ayre, published in 1833, for some illustrations of a similar fact.

As a useful preliminary to the examination of details touching the question of Infection being the principal agent in the propagation of the Choleric Pestilence, we shall here take a rapid survey of the most prominent circumstances connected with the rise of the great Epidemic of 1817, and of its subsequent diffusion over the greater portion of the habitable world. Dr. Copland's account of its origin is as follows:

"Pestilential cholera first made its appearance in Jessore, a populous town in the centre of the Delta of the Ganges, and cut off the majority of those whom it attacked. It spread from the town in all directions, and reached Jaulnah, on the Madras side of the Indian peninsula, in June 1818, and Bombay in August of the same year. It continued to spread and to prevail throughout all parts of India and the adjoining countries, and still prevails in many districts, although in various degrees of severity, &c., with intervals of complete immunity from its presence. Indeed, it may be said to have become naturalized in India, forming one of the diseases of the country."

He then tracks the subsequent course of the Pestilence in successive years; *eastward* to the Burmese empire, the kingdom of Siam, China, the Phillipine and other islands in the Indian Archipelago; and *westward* to Persia, Arabia, Syria, Judœa and the Georgian frontiers of Russia. In 1823, it had reached Astracan on the banks of the Caspian; beyond which it did not extend for the present. For five or six years subsequently, we know little or nothing respecting its progress; the pestilence seems to have been lying dormant in the regions to the southward of this great inland sea. All that we can say, with any degree of certainty, is that, in 1829, it suddenly broke out with great violence at Orenburg, a Russian town on the Tartar frontier, about 400 miles north of the Caspian and about 1000 miles distant from the places where it had prevailed extensively in 1823. It is universally admitted that no satisfactory explanation ever has been given of the source or causes of the unexpected outbreak at Orenburg: this is a point which seems to be conceded by all who have enquired into the subject. The pestilence continued in that town until February 1830, after which time it seems to have entirely ceased for several months in the Russian territory. In July, however, of this year, it appeared a second time at Astracan with intense malignity, destroying in twenty-seven days upwards of 4000 persons in the town, and of 21,000 persons in the province. It then ascended rapidly the Volga, and reached Moscow in September. It continued to spread westward and northward through Russia and Poland; also to Moldavia and Austria. In May 1831,* it reached Riga and Dantzig; in June and July, Petersburgh and Cronstadt; in August, Berlin; subsequently Hamburgh, and at length, in October, it appeared upon our own shores at Sunderland. London was not visited until the second week of February in 1832. The disease appeared at Calais a month subsequently, and, a fortnight later, Paris was visited by the pestilence. In June of this year, it made its appearance at

---

* In this year also, it prevailed in Egypt epidemically at Cairo, where it proved very destructive; also at Smyrna and Constantinople.

Quebec and Montreal, and also in New York. In July, it spread to Philadelphia, and several other cities; thence to nearly the whole of North and South America. It was not until the latter part of 1833, that it reached Spain. It visited several parts bordering on the Mediterranean in 1834; and reappeared, in a very partial manner however, in London and some other places in this country, as well as on the Continent and in North America, in the same year. In 1837, it proved very destructive in Rome. "It spread," continues our author, "to various other countries not mentioned in this brief sketch between the years 1831 and 1837; and few places were entirely exempted from it, excepting those which were placed under strict quarantine." It is much to be regretted that Dr. C. has not particularised the places that enjoyed the immunity to which he here alludes.

So much for the geographical history of the world-wide diffusion of the great pestilence of the 19th century—a history which will not fail to suggest, to the mind of the attentive reader, various reflections on the much vexed question as to what agency this diffusion was chiefly attributable. But we shall not say more upon this point at present; for we must recall the reader's attention, for a few moments, to the locality or region whence the pestilence issued. We have seen that Dr. Copland, without hesitation, asserts that "it first made its appearance in Jessore, a populous town in the centre of the Delta of the Ganges." The reader might naturally suppose, from this simple and unqualified announcement, that the truth of the statement had never been questioned, and that no doubt has ever existed as to the exact spot in which the pestilence was generated, and from which it emanated to devastate the earth. Now, what do we read on this subject in the Calcutta Medical Report, that was drawn up with such elaborate care, and after the most sifting examination of a vast amount of evidence?

"It is certain that nothing could be more erroneous than this notion of the local origin of the Epidemic. For, not to speak of its frequent occurrence so early as May in some parts of Nuddea and other districts already adverted to, it is quite clear from the statements of the medical staff, written separately and without interchange of knowledge or communication, that, more than a month previously to Jessore's becoming affected, the disease had begun to prevail epidemically in the distant provinces of Behar and Dacca; and that, before the expiration of the first week in August, it had firmly established itself in many other parts of Bengal."

After giving a variety of details, which it is not necessary here to record, the editor of the Report continues:—

"These facts are more than sufficient to shew the fallacy of every theory, which attempts to derive the disease from any local source; or to trace it to any one particular spot, as the centre from which it was emitted to the surrounding countries. They prove, without the possibility of dispute, that it broke out in very remote places at one and the same time, or at the distance of such short intervals, as to establish the impossibility of the pestilential virus being, in this state of its progress, propagated by contagion (infection), or any of the other known modes of successive production; and that its general diffusion was therefore referable to some causes of more universal operation."

How can we resist such testimony as this, based too, as it is, on the most ample and satisfactory evidence obtained in the very region where the epidemic took its rise? Is it not rather singular that our author should not have even alluded to it? One might almost fancy that he supposed that the admission of the disease having sprung up in various places about the same time was somehow opposed to his favourite tenet of its being a new distemper, essentially different from any endemic disorder of the country in which it arose. But, however this may be, there cannot be a reasonable doubt, we should suppose, that the epidemic of 1817 did not commence in any one single and definite spot. Mr. Orton, whom we shall afterwards find that Dr. C. is happy to quote as an infectionist, has distinctly asserted that it was in the district of Nuddea, and not of Jessore, that the disease commenced. "It is, however, shewn," says this most competent witness, "that this was far from being *its sole local cause*..... Such and so striking being the circumstances attending the rise of the malady, and its first and principal ravages all over lower Bengal, we may fairly infer that *it was owing to this exaltation of the common causes of endemic or sporadic disease that it took on the epidemic and contagious form*, and thus became capable of diffusing itself far and wide over the earth." A very important admission from an author whom Dr. Copland quotes with deservedly high commendation.

Dr. Johnson also, writing in 1832, expressed his opinion respecting the origin of the Epidemic Cholera in the following terms:

"It is clear to demonstration that the disease did not originate in Jessore; on the contrary, there is as good, indeed better, reason to suppose that it was carried to Jessore, than that it *first* broke out there. In truth, there is no proof that it sprung up in any one town, or even district; but, from some causes of which we are, and ever shall be entirely ignorant, it was generated in the province of Bengal, in several places at the same time, and very probably under similar circumstances. Let what will have been its origin, it did *not* commence at Jessore, nor do we know at what place it did commence, and consequently any argument or train of reasoning founded on such assumption is utterly baseless."*

It would also be leaving a very inaccurate impression on the reader's mind—one, too, at variance with the history of almost all other devastating pestilences—if he were to suppose that the great Indian Epidemic of 1817 broke out all at once, and without premonition, "like a thief in the night." It was not so. The preceding year (1816) had been unusually sickly. An aggravated form of Remittent Fever prevailed epidemically in the upper provinces of India, and occasioned such mortality as "surpassed anything on record in the medical annals of Bengal." In many native villages the whole population was ill, and shops were shut for want of people to attend them; the banks of the rivers were at all times covered with the dying and the dead; in Cutch and in Scinde, several cities were said to be so depopulated that the living were unable to bury the dead. It would seem, also, that great mortality prevailed among the horned cattle.*

The Spring and Summer of 1817 presented singular deviations from their ordinary course. The rains set in earlier than usual, and the season

* Medico-Chirurgical Review, No. 33, p. 78.
* *Vide* Medico-Chirurgical Review for July 1832, p. 74.

was altogether unusually wet. During the first six months of this year, the endemic Cholera morbus had shewn itself sooner, and prevailed more extensively, than in former seasons.

The work of Mr. Orton, and also the Calcutta Report, give the fullest information as to the exceeding insalubrity of the year 1816 and beginning of 1817, throughout Bengal. The details will be found exceedingly interesting by every one who wishes to make himself thoroughly acquainted with the entire history of the great epidemic of this year.

It will be afterwards seen that the outbreak of the disease in other countries was very generally preceded by a greater amount than usual of sickness, more especially of diarrhœa and other bowel complaints, among the inhabitants.

We are now ready to examine the evidence adduced by our author in proof of his position that Infection has been the principal agent in the diffusion of the Pestilential Cholera. In commencing his investigation of the subject, he assures us that, from the very first, " his mind was entirely unbiassed, and desirous of adopting that view of it which well-ascertained facts should most fully support." We doubt it not; and all that we ask of him is to concede the same degree of candour and honest intention to those who may differ from him.

He first of all appeals to the evidence contained in the three official Reports from Bombay, Calcutta, and Madras—so justly characterised by him as the " original sources for information as to this and various other topics; because the opinions of the Indian reporters were generally derived from an extensive and varied experience of the disease during a number of years, and they were certainly not biassed in favour of contagion (infection)—that being a property which the diseases of India seldom present"—to which we have already so often referred. All these, he remarks, " favour the infectious nature of the disease more or less." He goes on very frankly to admit that " it is quite true that a majority of the surgeons and assistant-surgeons in India, who sent reports to their respective medical boards, state that they do not believe the disease infectious;" but then, says he, " a large number of them give a very different opinion, whilst the reasons, assigned by many for believing the disease to result from other causes than infection, are actually favourable to the existence of an infectious property;"—a remark, by the bye, not very complimentary to the sagacity of those gentlemen who witnessed the facts which they undertook to commemorate. But let that pass; and let us now look with 'unbiassed' minds at the more important statements in the three official reports to which so much importance is justly attributed by Dr. C.

That from Bombay was published in 1819, that from Calcutta in 1820, and the one from Madras in 1823. The members of the Medical Board in the first of these Presidencies, after candidly admitting that " they are aware of the doubtful nature of the ground on which they tread," adopted the following conclusion, which, we need scarcely say, is prominently brought forward by our author.

" It appears to them incontrovertible that this disease is capable of being transported from one place to another, as in cases of ordinary contagion or infection, and also to possess the power of propagating itself by the same means that acknowledged contagions do—subject, however, to particular laws, with which we may never become acquainted—that is, by the acquisition of fresh material with which to assimilate."

The reporters, in another passage however, remark with great fairness and propriety, that the doctrine of the Infectiousness of the Cholera is "a question of the greatest importance and ought not to be hastily entertained as proved, nor rejected as unfounded; but prosecuted with that diligent enquiry and cautious induction which on every subject of science are so necessary to the attainment of truth: and we entertain a confident hope that the wide range through India which the disease has taken, will have afforded to some gentlemen more ample means of determining it than we possess." We shall see, as we proceed, the result of subsequent researches on this very point among the medical men of India.

After quoting the above general conclusion of the Bombay Board, Dr. C. next adduces the testimony of an unprofessional gentleman, which, as a matter of course, cannot carry much weight, and then that of several medical officers, whose evidence he considers to be highly favourable to the doctrine of the infectious nature of the epidemic. The following is a part of this evidence:

"Dr. Taylor reports, that 'whenever the disorder appeared in any particular spot or family, a considerable proportion of the family or neighbours were attacked within a very short period of each other; on many occasions I have seen three or four of a family lying sick at once.' Dr. Burrell informs us that, in the short space of six days, every attendant, in his hospital, on the patients affected with cholera had the disease. And Mr. Craw states, that every one of the attendants, 30 in number, in the hospital of the 65th Regiment, were attacked."*

Now, may it not be very reasonably conjectured that, in many of these instances at least, the disease was caught quite independently of direct infection from the sick, seeing that there prevailed at the time in the affected localities an epidemic disease, which is described by another of the reporters as "creeping from village to village, raging for a few days, and then beginning to decline." The very same sort of evidence might be adduced to prove the infectiousness of Influenza, if the mere circumstance of many members of a family lying ill at the same time be considered as anything like satisfactory proof; and if we are to attribute the sickening of every one, without exception, of the attendants in the hospital of the 65th regiment to direct transmission of the disease, then indeed must Cholera be vastly more virulently infectious than the very worst forms of Smallpox or Scarlatina. But that little importance is to be attached to the point so prominently urged by Dr. Copland is tolerably clear from the circumstance that Dr. Taylor—whose evidence has been adduced by him in favour of infection—expressly tells us that scarcely any of his hospital attendants were attacked. Some of the other gentlemen bear testimony to the same effect. Mr. White, for example, tells us that, of the assistants

* Neither of those gentlemen however, it is worthy of notice, expressed themselves convinced of the infectiousness of the Cholera. The former merely stated that "he would be cautious in reporting the disease not infectious;" while the latter was unwilling, he said, to express any decided opinion upon the point in question.

and dooly-bearers in his hospital, who used to assist the sick into and out of the bath, and in every other way, not a single one suffered. This gentleman adds, that he had "seen the disease affecting a particular part in one cantonment for days without reaching another part, although a constant communication was kept up between these parts all the while."

In conclusion, it is but right that our readers should be made aware of the fact, that out of 22 gentlemen who sent in reports to the Bombay Board, 10 expressed themselves as decidedly non-infectionists, and 3 only as infectionists; and of the latter, too, one was not a medical man.*

We now proceed to the Calcutta Report, which was published a year after that from Bombay, and was therefore based on a much larger experience. It was drawn up by Mr. Jameson, from the communications of a hundred medical officers in different parts of the Presidency. It is, perhaps, worthy of notice that this report differs from those of the Bombay and Madras Boards in the very important circumstance that, whereas in the latter two the individual communications, or large extracts from them, are given in detail, these being merely preceded by a summary of the evidence, in the first we have only an elaborate summary of their contents, consolidated and fused into a systematic treatise. Dr. Johnson remarked that each plan had its advantages, and that he was well pleased that both had been adopted. Dr. Copland, however, seems to think differently, and is very indignant with the Editor of the Calcutta Report, charging him with most culpable ignorance of the commonest laws of infectious diseases, with extremely loose and illogical reasoning from the data furnished to him, and with a preconceived partiality in favour of a particular side of the question at issue. It is to be regretted that Dr. C. is so vehement in his denunciation of almost everything that is opposed to his own views, as it inevitably leaves an impression on the reader's mind, that he is contend-

---

* To shew that even the most strenuous infectionists are not disposed to carry their views so far as Dr. Copland, we shall here quote a passage from a letter addressed by Sir Gilbert Blane to the Court of the East India Directors in 1825:

"Nothing, I think, can be more clear, from the very luminous history of Epidemic Cholera given in the Bombay Report, than that this disease has arisen, on various occasions, without owing its existence to contagion (infection), and without communicating it to others, as exemplified in cases of a very limited number of individuals, unconnected and uninfluenced by each other; in which circumstances, after a partial prevalence, the disease has disappeared without spreading, as stated in several passages of the Report; while it is equally manifest, from other parts of the narrative, that the disease was certainly contagious; nor is there in this anything contradictory or dissonant to reason and experience in analogous cases. For it is fully ascertained, with regard to the Typhus Fever of Europe and the Yellow Fever of the West Indies, that, though they sometimes appear in a sporadic and uninfectious form, they do also, *under certain circumstances, assume a form decidedly contagious:* these circumstances are chiefly crowding, want of cleanliness, and deficient ventilation, which add concentration and virulence to the venomous principle."

Here it is obvious that the doctrine of "contingent," not of intrinsic and "essential," infection is distinctly advocated by one of the great champions of our author's general views.

ing rather for the triumph of his own opinions than for the discovery and establishment of the truth. Mr. Jameson, although decidedly opposed to the doctrine that the propagation of the Cholera was mainly attributable to infection, has, with a most exemplary candour, related a variety of circumstances, connected with the appearance of the pestilence in different parts of Bengal, that may be supposed to have an opposite bearing. For example, he says in one passage :—

"If, setting aside the circumstances militating against it, we take it for granted that the infection was truly received by the centre and Hansi divisions of the army from the detachments above-mentioned, we must believe that the disorder, although not communicable by *contact* from person to person, was so from one large body to another ;* and that, whenever the poison got a-head amongst a number of men, it assumed some new quality, so as, when mixed with the atmosphere, to become infectious. What constituted this additional quality, we cannot pretend to determine ; but, in support of its existence, we may quote the predilection of the epidemic for cities and camps ; the infection of the left division, and the Nagpore and Meerut troops, immediately after entering into the diseased medium at Jubbulpore, Nagpore, and Delhi, and the similar case of the troops and followers in attendance upon the Governor-general being attacked shortly after communicating with an infected village in the Gurrockpore district."

Dr. Copland seems to be quite astonished that any one acquainted with these and such like statements should hesitate, for one moment, in his opinion as to the infectiousness of the disease. He regards them as perfectly conclusive, and expresses indignant astonishment that Mr. Jameson, *when* in possession of such information, should have been allowed by the Government to proclaim his belief that infection was not the principal agent in the propagation of the pestilence. The dispassionate investigator of epidemic diseases, however, will not be so easily satisfied, nor be willing to commit himself to such a hasty and peremptory conclusion. Our author indeed alludes to still more convincing evidence in favour of his views, contained in certain documentary papers at the East India House in London, which he had the opportunity, he tells us, of inspecting in 1827 ; but, as he does not communicate any part of their contents, he cannot expect that much importance be attached to the statement.

Dr. Copland's treatment of the Calcutta Report throughout is so very partial and one-sided that it tends to shake, a good deal, our confidence in his fairness in the way of quotation from the authorities which he has consulted. Many of the most important facts and reasonings adduced by Mr.

---

* It is pretty clear, from this and other passages in the Calcutta Report, that Mr. Jameson has used the word *contact* erroneously; viz. to denote merely proximity to, and immediate intercourse with, the sick ; for, in what other sense could he have spoken of one large body of men being in contact with another ? Indeed, he has himself told us that this was his meaning; yet it is upon such a passage as the one here quoted, that Dr. Copland accuses him, that "in all his remarks, he seems to suppose that contact is requisite to the propagation of contagious disease." And is it not so ?—the reader may naturally enquire; if the word *contagion* is to be used in that very sense laid down by our learned author himself, to denote "infection by immediate or mediate *contact*—as a pollution by the touch."

Jameson are passed over without even so much as the slightest notice. Thus, for example, he does not allude to the highly-important circumstances of the striking immunity on many occasions of particular bodies of men in the midst of the disorder, or having the disorder in the midst of them; of the disease having been often altogether absent in places and villages intermediate between two towns where it prevailed, although constant communication existed all the while;* nor of troops being quickly affected with the pestilence when they were brought into, or made to pass through, pestiferous districts, and of it as quickly subsiding when they were withdrawn from them.† He makes no allusion to that particular habitude which the disease so generally manifested of running a regular and definite course of outbreak, increase, maturity, decay and cessation within a very short space of time—say, from two to three or four weeks.‡ He does not tell us that

---

* "Let the reader call to mind the innumerable detached spots which remained free, when all around was sickly, and remember that, in no single case, was any restraint placed on free intercourse between the healthy and diseased; and he must come to the conclusion that the epidemic was totally independent of the common laws of contagion."

† The description given by Mr. Jameson of the terrible outbreak of the pestilence in the camp of the Marquis of Hastings on the banks of the Sinde is full of painful, but instructive, interest. Nearly 9000 persons were carried off in the course of one week; the disease rapidly subsiding and ceasing when the troops were withdrawn from the locality where the pestilence broke forth. The Govenor-General thus spoke of this visitation of his army:—"The dreadful pestilence, which made such havoc in the Division under my immediate command, forced me to quit the banks of the Sinde, and to seek a more favourable country for the recovery of my numerous sick. I did not find this until I was fifty miles from the river which I quitted. Fortunately, the change of air was rapidly beneficial."

The memorably destructive outburst of Epidemic Cholera, in the course of last summer, at Kurrachee is a similar instance of an almost instantaneous invasion of the pestilence. In the course of little more than a week, it carried off upwards of 8000 human beings! Mr. Orton also has described a sudden and awful eruption of Cholera in a marching troop, succeeded by a terrific thunder-storm: p. 377 of his work.

These terrific outbreaks can be compared, in their effects, only to the breath blast of the Simoom in the desert. Infection can have nothing to do with the dreadful mortality of such visitations. How admirably has the poet described the destruction of the host of Sennacherib by the "pestilence that walketh by noon-day and the arrow that flieth by night!" There is as much philosophy as truth in the description.

"Like the leaves of the forest when Summer is green,
That host with their banners at sunset were seen:
Like the leaves of the forest when Autumn hath blown,
That host on the morrow lay withered and strown.
For the Angel of Death spread his wings on the blast,
And breathed in the face of the foe as he passed."

‡ This is a very characteristic feature of epidemics in general. Dr. Johnson very soundly remarked, in reference to this point: "It is certain that this law of Cholera is not absolutely conclusive against its infectiousness; but, inasmuch as it constitutes a most essential difference between Cholera and diseases manifestly infectious, it serves to destroy the analogy between them, and makes us hesitate in imagining that their propagation can be governed by the same laws."

the natives of India have never regarded the disease to be of an infectious nature; although he availed himself of this very argument—that of the prevailing popular belief—in support of his opinion that the epidemic of 1817 was a new disease, altogether different from the endemic cholera of the country. He has also omitted to state that "the whole body of the medical officers in Bengal, who had an opportunity of seeing and remarking on the disease, without a dissenting voice, concur in declaring that it is not contagious (infectious)."* Now what, we may fairly ask, were the circumstances which produced such unanimity of opinion among those who, being on the spot, were surely better fitted to judge of the nature of the then-prevailing disease, than any gentleman, however learned, who was never in India in his life-time? The Report has told us in these words:

"The opinion of the medical observer was, in all situations, founded upon his noticing the following facts.—In his attendance upon patients labouring under the disease, he did not find himself, or his assistants, more liable to be attacked by it, than such persons as had no communication with the infected.† He could not attribute his escape to the effect of precaution, for he took none; nor to the limited nature of his intercourse with his patients; for the disorder, and the remedies employed in it, were such, that he was obliged to be constantly handling the body of the sufferer, and could not with safety leave his bed-side during the height of the attack.—It might even be said, that in every case the patient and he breathed upon one another; so that, had the effluvium, exhaled from the lungs or skin of the patient, been truly infectious, even in the slightest degree, he could have had no chance of escaping.—Next he saw that, where one member of a family was ill, the others were not more liable to get the disease, than an equal number of individuals picked out from the general body of the community.—This must be taken with some allowance.—Sometimes two or more members of one family were seized; but in such cases, they were generally all taken ill together; were living in the same unwholesome situation; and had been previously exposed to some manifestly strong exciting cause; as the eating of noxious food; sudden vicissitudes of temperature; and the like.—In the rare instances, in which one fell ill at some distance of time after another, if we do not choose to consider the occurrence as purely accidental, we shall be at no difficulty to explain it, upon remembering the depressing influence of fatigue, fear, sympathy, and grief—all powerful predisposing causes. * * * * In no one instance were the dooley-bearers, native compounders, or any other part of the large hospital establishments then necessarily kept up—although all were often so hard worked as to be scarcely able to stand from fatigue—more sickly than other descriptions of followers; nor did the soldiers, who constantly flocked

---

* "To this unanimity of conviction there was originally one exception; but, from more extended experience, that individual has since modified his opinion." —*Bengal Report.*

† This a very striking fact. From a medical list, consisting of between two hundred and fifty and three hundred individuals, most of whom saw the disease largely, only three persons were attacked, and one death only occurred. The fatal case took place at Barrackpore, a station very little visited by the epidemic; the two others, which were nor severe, occurred in the Centre Division of the Army. There, too, one of the Surgeons of His Majesty's Corps was cut off; the only medical officer belonging to the King's service known to have been affected. In Nagpore the Medical Staff remained for several days, night and day in the hospitals, and yet all escaped."

to the hospitals, to see and watch over their sick comrades, appear by that means, to be more susceptible than others, of the disease.—Nor were those patients, who were ill of other disorders, although always surrounded by persons in every stage of Cholera, therefore more liable to be attacked."

Other arguments might be adduced from the above Report which strongly militate against Dr. Copland's peculiar opinions; but our limits prevent us from doing more at present. The reader will now be able to judge for himself whether this Report can, anyhow, be said to "favour the infectious nature of the disease," as alleged by our author.

The present is perhaps as favourable an opportunity as we may have to allude to a topic in the history of Epidemic Cholera, on which there has been some difference of opinion—we mean the liability, or not, to a second attack of the disease. Upon this point, Mr. Jameson says:—

"Another curious circumstance in the economy of the disease, was, that not only were persons, who had once undergone its attack, free from its further assaults; but even individuals, and bodies of men, who having come within its pestilential influence, had escaped unaffected, were nevertheless much less obnoxious to its future visits, than those who had not before been exposed to the virus. In other words, a village, which was visited by the Epidemic during the first year of its prevalence, would, on the disease re-appearing in that part of the country, be much less likely to suffer than another village, which had not before been affected: and an individual, going from the former into the infected air of the latter, would have a better chance of immunity, than its inhabitants, who had not undergone the previous seasoning. This was the case to a greater or less degree in every part of the provinces; in which it was generally remarked that the Epidemic, on its recurrence, either did not at all revisit the places formerly affected, or only in a much lighter manner, than those to which it was yet a stranger."

Other writers, however, have expressly told us that one attack does not confer immunity from a subsequent seizure. Of this number are Mr. Scott, Mr. Hamilton Bell, and Dr. Budd. Messrs. Russell and Barry have said that relapses were very unfrequent; and Mr. Orton has expressed a similar opinion.

We shall now notice very briefly the evidence of the Madras Report, which was not published till nearly four years after the one from Calcutta. We have already stated, in a previous page, that it is altogether opposed to our author's opinion of the epidemic of 1817 being an entirely *new* disease. Let us now see what bearing it has on the question of Infection being the principal agent in its dissemination. Any one reading Dr. Copland's notice of the general Report, might suppose that Mr. Scott, the Editor, as well as the most experienced and able of the medical officers who sent in communications to the Board, were decided infectionists. That the extracts, adduced by him from the individual reports of those gentlemen, strongly favour the opinion of the infectiousness of the disease, no one will deny. But why has he limited his attention entirely and exclusively to those statements only, which favour one side of the question? Is this the way to arrive at truth, or to impress the reader with confidence in the impartiality of an author of a great work of professional reference?

Mr. Scott has himself told us that, "if this question could have been decided simply by the opinions of a majority of medical men, it would have been already set at rest against the doctrine of contagion or infection;" "for there are few subjects, perhaps, on which so little diversity of sentiment has existed." And he adds:—"It is not contended, by those who embrace the doctrine of Infection, that Cholera has not arisen spontaneously, as well at the present as in former times; nor is it considered that this circumstance affects the question." Most true; it does not affect the question whether the disease ever manifests an infectious character; but it most vitally affects the question whether Dr. Copland's opinion, that infection is the principal agent in its dissemination, can be reconciled with the history of the epidemic. Without saying more upon this point, we shall only remind the reader that Dr. Johnson has given, in the last two editions of his work on Tropical Climates, a tabular analysis of the individual reports—amounting in all to 65—from which the general report has been constructed.

As respects the question of the infectiousness of the disease, we find the following summary at the end of this analytic table.

| | | | |
|---|---|---|---|
| " 1 | Suspects it to be contagious. | 13 | Decidedly not contagious. |
| 1 | Doubtful. | | |
| 1 | Contingent contagion. | | |
| 1 | Endemic and contagious. | | |
| 1 | Slightly contagious. | | |
| 1 | Inclined to think it contagious. | | |
| 1 | Sometimes communicable. | | |
| 1 | Decidedly contagious. | | |
| 2 | Infectious. | | |
| 10 | | 13 | |

42 make no allusion to contagion. Of these 42, the great majority speak of the disease as an epidemic arising from some atmospheric or terrestrial cause; and, as the queries of the Madras Board particularly directed the attention of medical officers to the subject of contagion, we may fairly conclude that, when no allusion to contagion is made, no proofs of it presented themselves to the observers. Mr. Scott, the Secretary of the Board, sums up the opinions of the contagionists and anti-contagionists, and gives no opinion himself on either side."

So much for the evidence on the important question of the infectiousness of Cholera, to be derived from the three Presidential Reports. We leave it to the reader himself to decide whether he will agree with Dr. Copland in the following propositions:—

"I have now shown, from the chief sources, that the disbelief of infection, in respect of the pestilential cholera, was not general in India—that the productions which issued from the three Medical Boards very strongly favoured, and indeed proved, the existence of this property,—that two out of the three actually insisted upon the activity of its influence,—and that, therefore, the dangerous opinion, so very generally propagated, and even acted upon, both in this and foreign countries, that the authorities of India did not consider the disease infectious, is entirely without foundation in truth."

Before taking leave of the important branch of testimony furnished by writers who witnessed the epidemic at the time of, and after, its outbreak in 1817, Dr. Copland briefly alludes to the published opinions of three gentlemen who have written upon the subject—viz. Mr. Orton, Mr. Annesley, and Dr. H. Kennedy. The first he quotes as a decided (!) infectionist;* the second, as one who ought to be, although he has declared himself otherwise; and the last as an infectionist, who "justly places particular stress upon the peculiar odour exhaled from the bodies of the affected, as indicating the generation of a principle calculated to propagate the malady!"† He has not considered it necessary to allude to any other of the numerous writers who have recorded what they saw of the disease in India. This omission is surely much to be regretted; it seems scarcely fair either to those gentlemen who have benefited the public by making known their experience on an important subject, or to the reader who must certainly wish to be made acquainted with all the most valuable testimony upon it. Let us very briefly notice the names, at least, of some of the most competent witnesses.

We have already said that Mr. Annesley, a high authority certainly, is a non-infectionist. "The limitation of the disease," says he, "to places where there existed no natural obstacles to its extension, militates most conclusively against any idea as to its being a contagious (infectious) disease, and seems to point to the existence of some difference in the quality of the atmosphere." Dr. Whitelaw Ainslie, a very experienced medical officer in the Company's Service, expressed a similar opinion. He has distinctly stated that Epidemic Cholera does not differ, in essential pathological characters, from the Sporadic disease; but only in *grade*, depending

---

* The work on Epidemic Cholera by this gentleman is certainly a very able and instructive one. He is of opinion that some of the pestilences which devastated Europe in the 14th and 15th centuries—especially the sweating sickness—were almost identical with Cholera. While he advocates the infectiousness of the disease, he candidly admits that "contagion (infection) alone is inadequate to the production of an epidemic disease;" and that "there is little, if any, increase of danger from the most intimate communication with the sick during the prevalence of the disease, above that which attends the common intercourse of society:"—that, "in Bengal, not more than three medical officers out of the whole list were known to have been attacked up to 1820, and but one died, although nearly the whole of them had largely witnessed the disease;"—that, "although the importation of the disease has so often been found immediately to precede its appearance in the inhabitants of a place, and even the first case to arise in the neighbourhood of the imported virus, it has scarcely ever been possible to trace these cases to personal communication in anything like a regular series, as may generally be done with the plague and other decidedly contagious disorders." Mr. Orton, we may remark in conclusion, believes that "it is by means of *fomites* that the disease is, in a great measure, propagated over a country."

† This gentleman, the 2nd edition (recently published) of whose "Notes on the Epidemic Cholera" was noticed in the number of this Journal for January last, has not, we may remark, adduced a single instance from his own observation of the infectiousness of Cholera. After quoting from the reports of others, he closes his remarks with these words: "I know no character, belonging to any contagious disease, which Cholera does not appear to me to possess; and if it be not contagious, I know no other disease which I should be inclined to consider so"! Although an infectionist, Dr. Kennedy does not agree with our author in regarding the Pestilential Cholera as a new disease: "there is nothing to surprise us that this disease raged as an epidemic 40 years ago." This was written in 1826.

on various causes, of the nature of which we are ignorant.* Dr. Smith has stated, as the result of his observations in India, that no instance ever came under his notice, where the diseased person infected another; and the immunity of the attendants, as well as of the friends of the sick, who often crowd around the death-bed of their unfortunate comrades, sufficiently proves that the disease is not propagated either by contact or by atmospheric infection.† Dr. H. Young, who was in Calcutta when the pestilence broke out there in 1817, has expressed his strong conviction "of the non-contagious nature" of the disease.‡ Mr. Corbyn says; "from personal experience, I feel satisfied that the notion of the Cholera being contagious (infectious) is quite unfounded."|| Unquestionably one of the ablest writers on Epidemic Cholera is Mr. G. Hamilton Bell. His work§ was universally acknowledged to be a most valuable production, based on very ample experience, and replete with an immense amount of information. He served in India from 1818 to 1827, and, from his position as residency surgeon at Tanjore, possessed unusual advantages for watching the disease under every aspect. Dr. Copland has perhaps done wisely not to grapple with either the facts or the reasonings so powerfully enforced in Mr. Bell's work. It is there emphatically asserted, that "Cholera Asphyxia is not a new disease to those natives (of India), but seems to be in many places almost endemical;" and that "it has been repeatedly ascertained that Cholera patients may be carried into hospitals crowded with patients labouring under other diseases, without these or the hospital attendants having the disease communicated to them." Mr. Searle also, who saw much of the pestilential Cholera both in India and in Europe, has spoken to nearly the same effect.¶ He attributes the disease to the operation of a malaria, aided by a peculiar condition or epidemic constitution of the atmosphere. But it is unnecessary to enlarge our list of authors who have published accounts of their experience of Cholera in the East. Suffice it to remind the reader that the late Dr. Johnson—than whom never was a medical writer better fitted to sift evidence and enucleate the truth—as well as Mr. Martin, whose authority on tropical diseases is deservedly so high, have uniformly rejected the doctrine so warmly espoused by Dr. Copland, that infection is the principal, or even an influential, agent in the diffusion of the disease.

Such then being the case, we cannot help saying how much surprised we were on reading the following passage—more remarkable certainly for rhetorical embellishment than for scientific argumentation—in our author's narrative.

"Are we to expect these comprehensive views of the history and modes of

---

* *Observations on the Cholera Morbus of India,* 1825.

† *A few Practical Observations on the Spasmodic Cholera of India,* 1830.

‡ *Remarks on the Cholera Morbus.* 1831.

|| *Treatise on the Epidemic Cholera in India.* 1832.

§ *A Treatise on Cholera Asphyxia, or Epidemic Cholera, as it appeared in Asia and in Europe,* 1831.

¶ *Cholera, its Nature, Cause, and Treatment.* 1831.

propagation of a disease from those who have seen but a little, and described only what they have seen; or from those who dispassionately investigate the origin, the causes, the phenomena, and the relation of all that has been observed and recorded, and cautiously weigh the evidence on either side of a disputed topic connected with it? The captain of a company, or even a colonel, performs an important part, individually, in an army during a general engagement; but he can know little, personally, of the disposition, changes, and evolutions of all its parts, and of the plan of strategy, according to which it first acted, or was led to change its operations, in order to meet or counteract those of its opponent. Like the commander-in-chief of the whole army, we, who collect, compile, arrange, and digest facts, on both the one side and the other of a disputed subject—who observe closely what has occurred within the sphere of our own experience,—who compose, weigh, and meditate upon the whole evidence, personal as well as testimonial, with our minds uninfluenced by prematurely conceived ideas, are the best suited to investigate, and to conclude respecting them. Placed, by the number of accumulated facts, and by minds accustomed to view and to investigate the difficult operations of Nature, on the elevated table-land of human science, we may be admitted to be more able to take in a comprehensive view of the causes and nature of disease, and to come to accurate conclusions respecting it, than many of those who, as observation has shewn, have drawn hasty inferences from a few and very imperfectly investigated occurrences."

Quitting now the testimony of East Indian authorities on the important subject which we have under consideration, we must invite the reader to accompany us in examining the evidence, adduced by Dr. Copland and others, respecting the transported infection of Cholera, after the pestilence had left the limits of Hindostan, and had entered upon that westward course of mysterious advance which it pursued until it reached the shores of America. Our author's notice of this part of his enquiry is intentionally very brief; "because," says he, "the identity of the malady in both hemispheres having been fully and generally admitted, and its infectious nature in India having been completely proved (!), it must necessarily possess the same character in Europe, unless counteracted by powerful means."

That the greater number of the instances, enumerated by Dr. C., of the disease being *supposed*, or *believed*, or *considered*, or *said* (for he very properly avoids making any strong assertion) to have been conveyed to different places by vessels from infected parts, and of the exemption of certain other places from its invasion, in consequence of quarantine and other precautions, are of the most questionable authenticity, must be apparent to every one. What will the reader think of such *proofs* as these?—

"M. Hubenthal states that a peasant, having arrived from Arkatal, on the borders of Persia, at the village of Neskatshne, to visit an uncle, was seized upon the night of his arrival with the disease. The persons, engaged in restoring the heat of the body by friction, &c., four in number, were attacked on the following day, and three of them died. Precautions were taken by the police to arrest the progress of the pestilence in the village, and it spread no further"!

Here is another *proof*:

"Dr. Meunier states that, at Bagdad, where a third of the inhabitants was attacked, none were affected but those who approached the sick! Dr. Reimann says, that there was not a single instance of a town or village in Russia, which contracted the malady without previous communication with houses or persons affected"!

It would be altogether unprofitable to endeavour to arrive at anything like a satisfactory conclusion on the point at issue, by quoting the conflicting opinions of different writers as to the manner in which the pestilence was at first introduced into Astracan and other towns in that remote region. We should not, however, omit to allude to the evidence of Mr. Cormick, who was at Tabriz in October 1822, at which time the disease had reached the western boundary of Persia, and was steadily advancing in the same direction. This gentleman utterly repudiated the notion of the disease spreading by infection.*

When the Cholera appeared at Astracan in 1823, the Russian Government resorted to restrictive measures to arrest its progress.† "Whether or not these measures (we quote from Dr. C.) were the cause of its disappearance may be difficult to determine; but it did disappear, and it was not till 1830 that it shewed itself again in that city." Here, the reader will observe, there is a candid admission of doubt on the point mentioned; yet, strange to say, a few pages farther on, when combating the objections of the non-infectionists as to the utter inefficiency of any quarantine measures, our author does not hesitate boldly to affirm that "these succeeded for eight years in arresting its entrance into that place (Astracan)"! This is an easy way of settling a disputed point to one's own fancy.

We have already stated that the outbreak of the pestilence—after having been scarcely heard of for several years—in Orenburg in 1829, has been candidly admitted by almost every writer to be utterly unaccounted for. In the Russian Official Reports, Dr. Lichtenstadt has distinctly acknowledged that it was not possible to trace the invasion to any communication with infected places. The majority of the medical men in Orenburg, at first, denied that the disease was transmissible from one person to another; but several of them afterwards qualified this opinion, and declared for its infectiousness, although still professing their utter inability to explain how the disease arose, or whence it came. Dr. Lichtenstadt has informed us that none of the medical men, and scarcely any of the attendants upon the sick, were attacked. It is also well worthy of notice, that a malarious condition of the atmosphere prevailed at Orenburg during, if not before, the outbreak of the pestilence there; as will be seen from the following statement of Dr. Onufriev, physician of the circle or district: "During the prevalence of the Epidemic, there was scarcely a single inhabitant of the city who had not some symptoms of disordered digestion. One complained of oppression and pain in the breast; another of headache, slight sickness, looseness of the bowels and the like." This, we need scarcely

---

* *Medico-Chirurgical Transactions,* Vol. xii.

† Dr. Craigie of Edinburgh, who wrote so much and so ably on Cholera in the Edinburgh Medical and Surgical Journal, which, for many years, he conducted with distinguished ability, informs us that:—

"Each of these cities (Astrachan, Moscow, and St. Petersburg) was placed under a rigid system of seclusion and separation from infected places, by means of strong lines of military posts and barriers, which, in the case of Moscow, were two-fold. Notwithstanding these precautions, however, the disease appeared in every one of these cities, and without the possibility of tracing it in any instance to unequivocal importation."

repeat, is one of the surest evidences of the prevalence of an epidemic disease.

"The introduction of the pestilence into Astracan in 1830," says Dr. Copland, "was traced to a vessel which arrived from Baku, at that time affected with Cholera." But then the question at once suggests itself—whence came the disease to Baku? But it is needless to argue where we have no data that can be depended upon; and Dr. C. must excuse us for not attaching the slightest value to the evidence of the clergyman, which he quotes at great length, respecting the mode of its importation into a town of the name of Saratoff. He does not mention anything about its introduction into Moscow. Sir W. Crichton, we may remark, has related with marvellous exactitude, the precise course, step by step, of the onward-advancing pestilence from the shores of the Caspian to this city, but candidly acknowledges that it could never be distinctly ascertained who conveyed it into its bosom.* "When the Cholera reached Moscow"—we quote from the official Report of Dr. Albers, who was sent by the Prussian government to Russia—"all the physicians there were persuaded of its contagious (infectious) nature; but the experience gained in the course of the epidemic, has produced an entirely opposite conviction. * * * * During the epidemic it is certain that about 40,000 inhabitants quitted Moscow, of whom a large number never performed quarantine; and, notwithstanding this fact, no case is on record of the Cholera having been transferred from Moscow to other places; and it is equally certain that, in no situation appointed for quarantine, any case of the disease has occurred. In many houses, it happened that one individual attacked by Cholera was attended indiscriminately by all the relatives, and yet did the disease not spread to any of them. The nurses also, as well as the physicians, escaped." Now this statement is made by a gentleman who professes himself an infectionist; although he candidly admits that "the infection of the Cholera differs from the nature of all known infections, and seems to approach nearest to that of Typhus." We may observe that both Dr. Albers and Sir W. Chrichton most emphatically stated, that not a single authentic instance could be shewn of the disease being propagated by *fomites*, such as articles of dress or furniture, or indeed by any inanimate objects at all; and the committee of Russian physicians, established at Moscow by order of the Czar, gave in an official report to the following effect: "The members of the Medical

---

* In the communication of Dr. Walker from Moscow to the British Government, we find the following statement:—

"In Moscow, by far the greater part of the medical men are of opinion that the disease is not contagious (infectious), but produced by some peculiar state of the atmosphere, not cognizable by either our senses or by instruments; that this was proved by almost every person in the city feeling during the time some inconvenience or other, which wanted only the exciting cause of catching cold, or of some irregularity of diet, to bring on Cholera; that very few of those immediately about the patients were taken ill; that persons had put on the clothes of patients who were very ill or had died of Cholera, had lain in their beds, or even alongside of corpses, had bathed in the same water where very bad Cholera patients had been bathed just before, and that none of these persons were taken ill."

Council have been convinced by their own experience, as also by the reports of the physicians of the hospitals, that, after being in frequent and even habitual communication with the sick, their own clothes have never communicated the disease to any one, even without employing means of purification."

While the Moscow physicians were, almost to a man, non-infectionists, most of their Petersburg brethren at first adopted the opposite side of the question. Dr. Copland simply informs us that "the introduction of the pestilence into St. Petersburg is referred, by Drs. Barry and Russell, to the arrival of vessels from places on the Volga, where it prevailed;" and he goes on to state that, "in that capital, the infectious nature of the disease was shewn not only by the mode in which it was propagated in various quarters, and by its introduction into and extension through the prisons and hospitals of the city, but also by its exclusion from some places by a rigid insulation." Among other convincing proofs, he mentions an instance where the pestilence was confined to one side of a village by the street being barricaded on the side where it had not reached, and all communication between the two sides of the village being interrupted! This, is one of "the many facts of the same description now before me." It is somewhat strange that he has not alluded to the opinions of any of the permanently resident physicians of the Russian metropolis. The late Sir George Lefevre, physician to the British Embassy there, and who had very ample opportunities of seeing the Cholera, has stated, (in the pamphlet which he published at the time), that he had no rational grounds for believing it to be contagious (infectious). During the prevalence of the epidemic, a general indisposition pervaded the whole population. After alluding to the idea of the infectionists, that the disease had been imported by a bargeman, Sir George very shrewdly asks: "How was it that none of this man's companions, exposed to the same causes, should have been attacked also?"—and, then he adds, "When, upon enquiry, it was found that, within the space of three days, the disease broke out in a dozen parts of the town widely-separated from each other, the supporters of contagion awaited further evidence, and the anti-contagionists increased with the increase of the disease."

Again, how does the following fact, related upon the high authority of the French medical embassy that was sent to Russia to investigate the nature, &c. of the pestilence, accord with the doctrine of its spreading by infection?—Kristofsky island, which is situated in the centre of the populous islands of St. Petersburg, and communicates with them by two magnificent bridges, and with the town by a thousand barges, which bring every day shoals of people to enjoy the pleasant walks there, remained entirely exempt from the pestilence during the whole of the time that it prevailed in the town. Almost all the French players retired to Kristofsky, and not one of them suffered; while, out of the small number of their companions, who remained in the town, several died from the disease. The French reporters attribute the exemption of this island to the quantity of wood upon it, acting as a screen against the malarious air in the neighbourhood. Similar observations have been made in Italy on the subject of Intermittent Fevers; and Dr. Rush has remarked the same respecting Yellow Fever.

We must not forget to mention that the Russian government were speedily convinced of the utter inefficacy of any quarantine or other restrictive measures to check the diffusion of the Cholera, and that, ere long, it utterly abandoned all such vain attempts. Drs. Russell and Barry were, at first, of opinion that the disease was transportable by clothes and other material objects; but they became convinced of the fallacy of this notion. And here, before we further notice the advance of the pestilence into Europe, let us look for a moment at its invasion of Egypt.

In August 1831, the pestilence, which had raged at Mecca in the preceding June, broke out at Cairo with dreadful violence. The terror and desolation that ensued were truly alarming. Clot-Bey, who gave a very interesting account of the invasion in the *Annales de Medecine Physiologique*, has declared his conviction that the dissemination of the disease was not transmitted by infection from one person to another, but only conveyed through the medium of the atmosphere. The reasons he gave for this opinion were—1, the nearly simultaneous outbreak of the pestilence in distant parts; 2, the non-immunity of the harems; 3, the non-immunity of the ships in the harbour at Alexandria, notwithstanding the suspension of all intercourse with the shore; 4, the immunity of some villages and districts in free communication with infected places; 5, the paucity of attacks among the servants at the hospitals; 6, the frightful rapidity with which the pestilence reached its acmé of destructiveness (it acquired its maximum of intensity in four or five days, and, after lasting for nearly a month, quickly subsided altogether); and 7, the almost universal prevalence of the disease in some one of its forms among the inhabitants. Clot-Bey candidly admits that Cholera, like other epidemic diseases, may, under certain circumstances, acquire an infectious character.

Dr. Copland does not make any mention of the disease at Dantzic. We shall supply the omission by quoting from the Official Report of that experienced physician, Dr. Hamett, who was sent there by our own Government to make observations. The opening statement of his Report stands thus:

"It remains a problem to this day, in what manner the Cholera Morbus originated in and about Dantzic; certainly it is not proved to have been brought hither from Russia or Poland by men or merchandise; because no ship had arrived at Dantzic from any Russian port previous to its appearance, and the intercourse with Poland had ceased since the beginning of Winter. The first symptoms of cholera shewed themselves indeed in such a peculiar manner as almost to exclude even the suspicion of its importation; and it is reasonable to conclude, that the disease originated here in some manner that has, as yet, not been explained. This is corroborated by the statements of several physicians, viz. that cases similar to cholera had been observed previous to the arrival of any vessel from Russia; and that the weather had been so remarkably unsettled since the commencement of Spring, that malignant diseases might be reasonably anticipated."

But, not satisfied with recording his own observations, Dr. Hamett applied to several of the leading physicians of Dantzic for their opinions. These are given in full in his valuable report, and well deserve perusal. They unanimously repudiate the doctrine of infection.*

---

* The Substance of the Official Medical Reports upon the Epidemic called Cholera, which prevailed among the poor at Dantzic between the end of May and the beginning of September 1831. By John Hamett, M.D. p. 190. Highley, 1832.

From Dantzic we pass on to Berlin. Dr. Copland merely quotes the opinion of a young physician there, Dr. Becker, that " the disease was introduced into that city by the vessels navigating the river Spree, which runs through the city;" but his evidence is far from being satisfactory. Nothing is said of Vienna or Hamburg, nor of the opinions of the medical men in those cities as to how the disease came to them. Dr. Vivenot, one of the most experienced physicians of the Austrian metropolis, published an elaborate document at the time, to shew that the disease was certainly not propagated by infection; and, with regard to the appearance of the pestilence in one portion of the Emperor's dominions, Dr. Craigie has informed us:

" The example of the kingdom of Hungary is perhaps still more forcible. Never, perhaps, was such a rigid and perfect system of non-intercourse enforced as that which was instituted on the southern, northern, and eastern boundaries of Hungary, when the disease appeared in Poland. Every defile of the Carpathian mountains was guarded with strong military posts, and watched with the utmost vigilance. Neither human beings, goods, nor brute-beasts, except, perhaps, an occaional wolf or bear, could penetrate into the Hungarian territories, unless at the muzzle of the firelock or the point of the lance. Yet how vain these precautions proved, was evinced in the course of a few weeks. For the disease appeared on the Hungarian side of the Carpathian chain in the month of June, and spread most rapidly through the towns situate on the banks of the rivers of that well-watered country. It will not do in this case to say that the sanitary lines were broken in the ordinary sense. Broken they no doubt were; but by no human power."

The introduction of the pestilence into our own country is declared by Dr. Copland to have been " certainly owing to the clothes and bedding of sailors, who died of it at Riga or other northern continental ports, or during the voyage from those ports, having been too generally preserved and delivered up to their friends, upon the return of infected vessels to British ports." The authority, on which he hazards this bold assertion, is the information which he received from " two masters of vessels," who furnished him with " several proofs of a most incontrovertible nature." * * * * * * * These masters," we are informed, " were, conformably with the then prevailing opinion, persuaded that the distemper could not be propagated by the clothes of those who had died of it; but facts soon afterwards occurred, which demonstrated to them the propagation of the malady in this manner, as well as by direct communication with the affected." Such is the evidence on which our author rests his own opinion! He makes no reference whatsoever to the published testimony of Drs. Brown and Ogden, Mr. Greenhow, and various other medical gentlemen, resident in Sunderland and its immediate vicinity, who have most emphatically assured us that not a jot or tittle of anything like satisfactory evidence could be discovered as to how, and when, the pestilence was introduced to our shores. Dr. Brown, who has written ably on the subject, expressly declares that he had met with several cases, which exhibited all the features of malignant Cholera, one and two months before the arrival of any suspected vessel into the port of Sunderland. But it is scarcely necessary to enlarge upon this topic. In our simplicity, we had fancied that there was not now a single medical man in this country, who persisted in the doctrine of the importation of the pestilence among us by the foul clothes of sailors who had died of it. Dr. Copland does not take upon himself to say positively from what particular place the disease came to us; but he demonstrates the facility with which its infectious principle might be transported from place to place, by relating an occurrence which befel himself. It was this. After having spent some time in the first cholera hospital that was opened in Bermondsey, Dr. C. drove to Pentonville in an open carriage, and there visited two of his relatives. On his entrance into their apartment, they at once complained of an offensive odour proceeding from his clothes. He said nothing as to where he had been. Next day, they were both seized with the early symptoms of the distemper, from which they ultimately recovered with difficulty. "Precautions were taken against the further extension of the malady in this house, and no case occurred in the vicinity, until some months afterwards"! It was fortunate that our author's own household escaped, seeing that his clothes must surely have retained some portion at least of the pestiferous effluvia with which they were so highly charged. We presume that the preceding *proof* of the transmission of Cholera is the same with that which is recorded in a note in an earlier portion of the Dictionary, where the following, still more remarkable, instance of the infectiousness of the disease is recorded:

"During the prevalence of Cholera in London in 1832, a parrot, in the apartment of a person who had the disease, died with the symptoms of it. Due precautions having been used to prevent its extension to the rest of the family, no one else was affected by it. Some other birds, in different parts of the house, escaped."

We shall not detain our readers with noticing the observations of any private individuals with regard to the infectiousness or not of the Cholera, as it shewed itself in any part of our own country; but it may be worth while very briefly to allude to the opinions and acts of those, whose official position necessitated a public avowal.

In the course of the month of June 1831, while the disease had not yet extended further east than Dantzic and Cracow, Sir William Pym, our Superintendant of Quarantine, addressed a letter to the British Government, recommending, among other immediate measures against the introduction of the Cholera, "that an order be issued, prohibiting altogether the importation of every description of woollen rags," and suggesting, at the same time, that the College of Physicians should be called upon for their opinion "whether other goods deemed susceptible (such as hemp, and flax, wool, &c.) could be imported with safety, without quarantine purifications, from parts where Cholera prevails."

The College declared, in their report—which was signed by the late Sir H. Halford, and by Drs. Turner, Macmichael, and Hawkins—that "the disease is of an infectious nature, and therefore does require that all persons, coming from an infected quarter, should be placed under a quarantine of at least fourteen days;" but, as there was no proof that the disease has been propagated by means of inanimate objects, "we cannot bring," it is added, "ourselves to believe it necessary to adopt the fifth article (just quoted) of Sir William Pym's recommendations."

Within a week from the date of this letter, the President and Fellows addressed the Lords of the Privy Council, re-stating their belief that

"Cholera is communicable from one person to another," and now recommending, until further information could be procured, "that articles of merchandize admitted into this country from infected places should be submitted to the usual regulations of quarantine"!

The Government, doubtless not satisfied with the mere advice here given, required from the College a more detailed statement of their reasons on which this advice was founded. Accordingly, a long letter was drawn up, embodying chiefly the account given by Sir William Chrichton (who, be it remembered, expressly denied the transmission of the disease by inanimate objects) to which allusion has been made in a previous page.

The recommendations, we may remark, that were issued by the first Board of Health, in October 1831, were so extravagantly stringent—to the effect, for example of "immediate separation of the sick from the healthy"—"conspicuous marks on infected houses"—"rags, papers, old clothes, &c. to be burnt"—"all articles of food to be placed in front of infected houses, and received by one of the family, after the person delivering them shall have retired," with many other precautions of a like character—that within a fortnight from their promulgation they were cancelled, and a new Board, consisting of men who had witnessed the disease, was instituted. Thereupon, the public was informed upon authority that "Cholera is not more infectious than Fever,"—that "this disease seldom spreads in families, and rarely passes to those about the sick, under proper observances of cleanliness and ventilation," &c. &c.!

We shall not say more upon the operations of the second or Central Board of Health than merely to recal to our reader's memory that one of their last acts was to issue *a recommendation* to the hospitals of the metropolis—and consequently to all public institutions of the kind elsewhere—*to receive Cholera patients, in future, as they would patients labouring under any other disease.* Need a single word of comment be made upon this truly valuable advice?—an advice which we trust may be always acted upon on any future visitation, either of the Cholera or of any other form of pestilence.

From London, let us pass over to Dublin, and thence to the French metropolis, and see what the leading men in these distinguished seats of professional literature thought upon the subject in hand.

The Board of Health for Ireland, in one of the first documents which they issued (March 1832), candidly admitted that "they were not able to trace the disease to any communication, by which it might have been introduced into the neighbourhood of Dublin;"* and, soon after the breaking out of the pestilence in Paris, the following most important manifesto was published there:

"The undersigned physicians and surgeons of the Hôtel Dieu think it their duty to declare, in the interest of truth, that, although this hospital has received

---

* Dr. Bullen of Cork, in his pamphlet on the Cholera in that city—which, by the bye, was not affected for several weeks after the appearance of the disease in Dublin, although constant and uninterrupted communication existed between them—has told us that, "upon every enquiry, there are no grounds for supposing that cholera was imported into Cork."

the greatest number of persons affected with the cholera, they have not observed any circumstance which authorises them to suspect that the disorder is contagious. (Signed) Petit, Husson, Magendie, Honon, Sanson, Gendrin, Recamier, Dupuytren, Breschet, Gueneau de Mussy, Caillard, Bailly.—*Paris, March* 31, 1832."

Most of the other hospitals in the French metropolis followed the example of the Hôtel Dieu.

The Report, also, of the Academy of Medicine in France expressed a decided opinion against the infectiousness of Cholera. Allusion is made in this report to the majority of the residents in Paris experiencing, in a greater or less degree, the influence of the epidemic constitution.

Leaving now the shores of Europe, we must turn our attention, for a few moments, to those of the New World, where the pestilence made its appearance in the Summer of 1832. It has always seemed to us that by far the strongest argument for the views of the importationists is the circumstance of the disease having manifested itself in parts of the globe, separated from each other by thousands of miles of sea between, and this too, as it has been very confidently asserted by these gentleman, soon after the arrival of vessels from an infected port. It is therefore necessary to examine, with all possible fairness and impartiality, the proofs of the assertion in question. The two most remarkable instances of the alleged introduction of the pestilential cholera into regions, very far apart from each other by intermediate sea, are those of the Mauritius in 1819, and of Canada in 1832. The former is perhaps the more remarkable of the two; and, being also the earliest in point of date, as well as the least generally known, we shall first look at its history.

Dr. Copland—following the statements of Moreau de Jonnes and some other writers, chiefly French, on the subject—has ascribed the introduction of the cholera into the Mauritius to infection derived from the Topaze frigate, which had arrived from Trincomalee, where the disease, it is asserted, prevailed at the time of her departure.

Let us see on what grounds this opinion—which has been repeated by one author from another, without any circumstantial evidence having ever been adduced—rests. With the view of ascertaining the authentic particulars of the case, we have examined the original official reports sent home by our military medical officers then resident upon the island, and which are now preserved at the Army Medical Office here.* We beg most distinctly to assure our readers that the following statements are entirely derived from these documents, that we have added nothing and have withheld nothing; all that we profess to do being to give a brief abstract of their contents. The most perfect reliance may therefore be placed on what we set down.

A committee of English and French medical men was appointed by the Governor of the Mauritius, General Darling, to enquire into all the circumstances connected with the appearance of the cholera on that island. It is from the report of their deliberations, signed by Dr. Burke, the then chief medical officer there, that the following memoranda are derived.

---

* Our best thanks are due to Dr. Smith for the ready permission which he granted us of inspecting these reports.

In the course of the month of September 1819, a few cases, exhibiting all the characters of malignant Cholera, occurred in the private practice of one or two of these gentlemen: some of these cases proved rapidly fatal. The Topaze frigate from Trincomalee arrived on the 29th of October; and, on the following day, 30 of her crew, suffering either from Dysentery or Hepatitis, were landed and received into the hospital of the 56th Regiment: there was no case of cholera on board at the time, nor afterwards, while the frigate lay there.*

Numbers of negresses and other women were admitted on board, and there was free communication kept up with the shore. No case however of the disease occurred for upwards of four weeks after the arrival of the vessel; and then it occurred not in the hospital of the 56th regiment, but in the barracks at Port Louis. The first case in this regiment did not take place for two or three days later. After this time, the disease made its appearance in various places, and at length committed great and wide-spread ravages. It may be worthy of notice that, while the crews of many of the merchantmen suffered severely, not a single case occurred on board the Topaze. It is particularly remarked by Dr. Burke, that "none of the hospital attendants up to this date (19th Dec.) have been attacked." A similar statement is made by Dr. Kinnis, surgeon of the 56th regiment. During the pravalence of the epidemic, there was a sudden and unusually severe outbreak of the disease at a place called the Powder Mills; but, by withdrawing the soldiers from the locality, it soon subsided. Their com-

---

* As it is of primary importance to determine the sanitary condition of this vessel, before and after her arrival at the Mauritius, the following details, derived from the report of her surgeon Mr. Foy, will doubtless be read with much interest. The Topaze left Trincomalee with a number of her crew suffering from dysentery, diarrhœa, and fever. During the voyage, 17 of the dysenteric patients —63 in number—were seized with Cholera; and, of these 17, three died. Mr. Foy remarks:—"what has contributed to retard the recovery of the sick is a Scorbutic Diathesis, which has been for some time very general amongst the crew; to this I attributed the many cases of obstinate diarrhœa on board." He then proceeds to state that the whole mortality on board the Topaze, during the 18 months that she had been in commission, amounted to not more than 16 deaths. Of these, six were from Dysentery, five from Cholera, three from Hepatitis, one from Pneumonia, and one from Phrenitis. Mr. Foy never for a moment entertained the idea of the Cholera having been imported into the Mauritius by the frigate. Five days after his arrival there, he wrote to Dr. Burke officially, that "no contagious (infectious) disease exists on board, that none existed during the voyage, nor, to the best of my knowledge and belief, did any exist at the places we sailed from."

How do these statements agree with the assertions of M. Moreau de Jonnes in his semi-official communication to the French Government, two or three years later? He takes it for granted, that the disease was introduced into the Mauritius "par les communications maritimes;" and straightway he goes on to inform us that, "in the month of November, an English frigate arrived at Port St. Louis from Calcutta, having lost a number of her crew from a disease which raged on board."!

The circumstance of a disease like Cholera breaking out on board of a vessel at sea, and which had been quite free from it at the date of her departure from the last port, is a very interesting one. Might not the advocates of the Animalcular Theory of dissemination reasonably press it into their service?

manding officer stated to Dr. Burke that, "in many of the cases, the men had been griped and purged for several days before being attacked with Cholera." The pestilence continued to prevail at Port Louis and in different parts of the island until March in the following year, when it entirely ceased. The mortality amounted to about 8000,* of which above 1300 cases occurred in Port Louis alone.

Now, with respect to the question of the disease having been imported into the Mauritius and spreading by infection there, the following data will probably satisfy most of our readers. The closing sentence of the report alluded to above, and which is signed by the names of 14 French and 4 English medical men, stands thus :—" La Commission de Santé n'a aucun motif de croire la maladie regnante contagieuse, et cette opinion est unanime parmi tous les membres ;" and Dr. Burke, in transmitting the report to the Governor, uses these words :—" Both classes of the profession seem to be unaminous in not supposing the disease to be contagious (infectious) or of foreign introduction." Among the precautionary means recommended by the Commission, no allusion is made to quarantine or other restrictve measures.

We have omitted to notice that Dr. Burke distinctly states that the weather at the Mauritius had been very unhealthy for many months before the appearance of the Cholera ; so much so, that the natives had anticipated a sickly season. Some of the cases seem to have been of the most malignant character ; for Dr. B. mentions that " he had been called to see some patients who, by their own report and that of their friends, would seem to have been struck as if by lightning." Dr. Kinnis alludes to the almost entire immunity of children from the disease, and to the very small proportion of women who were seized.

In conclusion, it may be worthy to state that, from enquiries which Dr. Burke made at the time, it appears that a disease, very similar to the then existing epidemic, had been known at the Mauritius so far back as 1775—at which period we know that a malignant epidemic cholera existed in India. This circumstance, although certainly not sufficiently authenticated, deserves at least to be mentioned.

What, then, is the evidence respecting the Cholera having been imported into the Mauritius by the Topaze frigate ? That a ship, with a crew of nearly 400 men, left her last port without a single case of Cholera on board, but with upwards of 100 on the sick-list from dysentery, hepatitis, and other tropical diseases ; that three fatal cases of Cholera occurred during the voyage ; that no other case took place before her arrival at the Mauritius at the end of October, or during her stay there ; that a few cases of the disease had been witnessed on the island in the month of September ; that no fresh cases occurred for three or four weeks after the arrival of the frigate, in spite of there having been a free and unrestricted communication between the ship and the shore ; and then, that the disease made its appearance not in the hospital to which her sick had been conveyed, but in another part of the town. Need a single word be added, in

* Dr. Copland says 20,000. This exaggerated statement was first made, we believe, in the Quarterly Review.

the way of comment or remark, upon this simple enumeration of *facts*, the entire accuracy of which is authenticated, beyond all reach of doubt, by the written evidence of the medical men, civil and military, resident upon the island at the time?

With respect to the introduction of the disease into the neighbouring island of Bourbon, Dr. Kinnis states, in his report (the date of which is March 1820), that, "after running its course here, it appeared at Bourbon, in defiance of the most vigorous quarantine;" and Dr. Collier, who succeeded Dr. Burke as chief medical officer at the Mauritius, and acted as the head of the quarantine establishment there, mentions in his report, dated some years subsequently, that "Governor Mylius did not succeed by quarantine in shutting out the disease from the island of Bourbon, but that it appeared there in February (1820), and spread there in spite of lazarettos and other precautionary measures."*

So much for the oft-cited case of the Mauritius. We shall now see whether the evidence of those medical men in Canada, who had the best opportunities of becoming acquainted with all the circumstances connected with the appearance of the cholera there, is more favourable to the importation doctrine so energetically insisted upon by Dr. Copland. The following data are taken almost entirely from a very long and carefully drawn-up report upon the subject by Dr. Stewart, Deputy-Inspector of Hospitals, then chief medical officer in Canada.

The first case of Cholera in Montreal occurred on the 9th of June, and in the person of an Irish emigrant who had arrived that day from Quebec, in the Voyageur steam-boat.† This person had landed at Quebec in perfect health, from a vessel in which one or two of the passengers only had been sick during the voyage from Cork; nor was there any cholera on board at the time of her arrival. On the following day (10th), another emigrant was seized, and "the disease appeared at the same time in distant and opposite parts of the town." It soon burst out everywhere. The first case among the soldiers in the garrison of Montreal occurred on the 12th (June); and, from this date to the 18th, there were 75 admissions into the hospital. In the course of the 19th, the regiment in which it appeared was moved from their barracks, &c. which were deemed un-

---

* We have had the pleasure of conversing with Dr. Collier—who, after a very lengthened period of active service abroad in the East and West Indies, is now resident in London—upon the subject, and we had the satisfaction of finding that his views are altogether in accordance with those which we have advocated in the present article. He is entirely opposed to the doctrine of importation by infection, and feels assured that the extension of a disease like Cholera is quite independent of personal communication. It is, perhaps, scarcely necessary to add that Dr. Collier—as indeed every other medical man who has been in the East—recognises no essential difference between the endemic Spasmodic Cholera of India, and the "Choleric Pestilence" of Dr. Copland.

† This steamer had left Quebec, on the preceding day, with such a multitude of passengers that she was obliged to put back and land a number of them there, before she could proceed on her voyage with the rest. It would seem that Cholera broke out that very night among some of those who had been landed, and who had taken up their abodes in one of the lowest and filthiest parts of the town.

wholesome, and encamped at St. Helen's. From this time, the disease may be said to have ceased in it almost entirely.* A great number of the men, however, were afterwards affected with diarrhœa and other premonitory symptoms; but, by these being promptly checked, there was no further extension of the pestilence among the troops. None of the officers or of the medical men, and scarely any of the hospital attendants, suffered. Few women, comparatively, were attacked; and children were almost entirely exempt. Nearly the whole population of Montreal experienced the influence of the epidemic.

In Canada, as in other countries, the season preceding the appearance of the Cholera had been marked by unusual sickness. Typhus and Scarlatina, of a bad type, had been very prevalent. There was also much blight, and a greater mortality than usual among many of the domestic animals. A report got abroad in the Spring that some cases of malignant Cholera had occurred at Chippewa; but it turned out that the disease, which had proved so rapidly fatal there, was a concentrated form of Remittent Fever. It appears, however, from the testimony of Dr. Robertson—one of the most experienced physicians in Montreal—that he had seen, in the month of April, several cases, in which (to use his own words) "the symptoms were exactly the same as those subsequently witnessed during the prevalence of the malady;" and another of the resident physicians confirmed the accuracy of this statement.

Dr. Stewart very reasonably concludes, from the circumstances now briefly alluded to, "and from the rapidity with which the disease shewed itself at Quebec, Montreal, and other places in the country, distant from each other, at about the same time, that the diffusion could not be well ascribed to the operation of personal communication." He speaks also of "the want of proof of any person having landed from any vessel, on board of which Cholera had existed on the passage, before the appearance of the disease in the country," as a circumstance that was quite opposed to the doctrine of importation. The only vessel up to that time, on board of which the disease was reported to have existed, was at the moment moored off the quarantine station, situated several miles below Quebec. It is a circumstance, too, that deserves to be noticed that a vast number of emigrants (it is said upwards of 30,000) had arrived in Canada from Europe, before the Cholera broke out.

Now, with respect to the sentiments of the resident medical men as to the mode of the diffusion of the Cholera, Dr. Stewart says: "In Canada, and, I believe I may say in North America generally, the majority is in favour of non-contagion;" and again, "the doctrine of non-contagion is most generally supported." His own opinion is, that it may acquire an infectious property under circumstances unfavourable to health: in other words, he is a contingent infectionist.† He expressly tells us that nothing

---

* The entire number of cases of Cholera in the troops forming the garrison of Montreal from the 12th of June to the 30th September, was 107: of these, 39 proved fatal. Eleven only of the 107 cases occurred after the 1st of July.

† He mentions several instances of particular houses or dwellings appearing to retain so strongly the pestilential miasms, that persons taking up their abodes in them became speedily affected with the disease.

had been witnessed in Canada, to warrant the suspicion of the disease being transmissible by *fomites*. One of the closing remarks of his exceedingly elaborate and very valuable report is, that "this pestilence would seem to bid defiance to all attempts to arrest its progress."

The Government of the United States, we should remark, had attempted, in the first instance, to prevent the introduction of the Cholera into their territory by quarantine establishments along their side of the St. Lawrence; but it was soon convinced of their utter inefficacy, and at once abandoned them. The first case or cases, that occurred in New York, could not be traced to any distinct source or cause of contamination. So the Cholera Gazette of Philadelphia informed us at the time. This official document alludes to the introduction of the pestilence into the New World in these terms:

"From the numbers of emigrants who about this time (June 1832) had landed at Quebec and arrived at Montreal from England and Ireland, a first impression was created that they had been the means of transmitting the epidemic across the Atlantic. A more close investigation into the facts connected with the commencement of the disease in those cities, served to destroy this supposition. It could not be traced to importation. The emigrants and lower classes of Canadians were attacked simultaneously in both cities."

It is unnecessary to do more than merely allude to the curious circumstance in the history of the Pestilential Cholera, that it did not visit Spain till the close of the year 1833, and beginning of the year 1834—in which year it also re-appeared in this country and in North America—nor Rome till 1837. How, pray, can we account for these seemingly most capricious outbreaks of the disease upon the theory of importation? Had the communications between France and Spain been so admirably guarded for upwards of a year and a half, that the distemper, which was raging in Paris in the Spring of 1832, was thereby kept from Madrid till the early part of 1834?—or how came it to pass that it took several years more to make its way into Italy? Was it shut out by the quarantines on the coast and military cordons on the land? But, without dwelling upon these vagaries in the history of Cholera, let us for a moment call the reader's attention to the curious circumstance of the singularly isolated development or appearance of the disease on board the Dreadnought Hospital Ship, in October 1837, of which so interesting an account has been given, by Dr. Budd and Mr. Busk, in the Medico-Chirurgical Transactions. Surely, no one can, with "unbiassed mind" read their narrative, without repudiating for ever the opinion of infection being the chief agent in the introduction and propagation of the malady. Yet, Dr. Copland clings to his favourite doctrine with the most devoted tenacity; but only by shutting his eyes against facts which cannot be gainsaid, and his good sense against reasonings which it must be most hard even for him to resist. That he is perfectly sincere in his own opinions, we doubt not for a moment; else, he would never have committed himself to such confident and authoritative statements as those with which the whole article abounds. For example, in closing his remarks upon the interesting subject which has chiefly occupied our attention, he lays down the following position as one which he has satisfactorily made out:

"This disease is never produced without the presence of a certain leaven or morbific matter, which, emanating from the bodies of the affected, and floating in the air, is respired by those about to be attacked. This is the clear and only inference, connected with its transmission, that can be deduced from the body of evidence now placed before the reader. Those, who argue against its transmissible nature, cannot shew, since the irruption of the pestilence in India down to its arrival in this country and transmission thence to America, a single instance of its appearance in any place without the previous communication with an infected place or persons, of a nature to propagate the malady."

In another passage, he does not hesitate to assert that the disease "has entirely avoided those who placed themselves altogether apart from the rest of the community;" and that it has been "barricaded in some towns and shut out from certain districts and streets."

Now, first of all, we would remark that the opening position here laid down must strike every one as very strange:—Cholera never produced without the presence of a poison proceeding from the bodies of the sick! How, pray, was it first generated? If, as Dr. C. supposes, it was a new disease in 1817,—produced, be it remembered, no one knows how—what is there to prevent its arising in like manner during other seasons, and in other places similarly situated? Whether the remark of Dr. Copland, that "there is no evidence to account for the generation of the cholera poison in the first instance, and there is as little of its reproduction *de novo* on subsequent occasions," is meant by him as an answer to this objection, we do not quite understand. However this may be, it is pretty obvious that Dr. C. is not inclined to push his views upon this point too far; for, in the very next page, we find him saying that "whether this principle (of infection) originated with the first irruption of the malady, or has been reproduced on numerous occasions subsequently—the disease which reproduces it proceeding from a very different cause—is a difficulty which will not be easily solved." With respect to the second position, the weight of the proof, it will be observed, is adroitly turned over by Dr. C. to the adversary, instead of his even attempting to shew how the pestilence reached a vast number of places which he has enumerated. But we have not far to go in search of evidence against his view of the question. Two pages further on from the passage just cited, we read:

"The non-infectionists argue that numerous instances of Pestilential Cholera have occurred, which could not be traced to exposure communication, direct or indirect, with those previously affected. *This may be the case in a few instances;* but how difficult is it to prove mediate infection, or that which takes place through the medium of fomites; and it may be asked, on how many occasions are persons liable to be affected by an infectious principle, without being able to account for the manner in which it took place, or to refer to the individuals whence it emanated, or to the media through which it was conveyed?" True, most perfectly true; and the obvious inference, therefore, must surely be, that such diseases are propagated in other ways besides that of infectious emanations direct from the bodies of the sick. We verily believe that our author stands almost alone among the members of our profession in the extreme and extravagant opinion which he has avowed, that "the extension of Pestilential Cholera in different countries has been entirely owing to the neglect of quarantine and other means of prevention." There is little chance of almost any one assenting to so wild a fancy as this; but there is a chance that some medical men might still recommend a partial degree of restraint, and the partial adoption of prohibitory measures (notwithstanding the utter failure of all attempts of the kind), in the event of a second invasion of Europe by the Epidemic Cholera—an event which seems, from the recent accounts of its progress westward, to be anything but improbable. This, indeed, has been the chief reason of our having devoted so much time and trouble to the investigation of the question upon the present occasion; seeing that, ere long, it *may* engage the thoughts not of the medical profession alone, but of the public generally. The authority of Dr. Copland is deservedly so high, and his great work is so generally known and esteemed, that it was nothing more than a duty to sift and determine the value of opinions, upon a subject too of such deep and universal interest, to which we knew a very wide currency would be given. Whether we have shewn cause for contesting their accuracy and disputing their validity, we gladly leave to others to decide; nor do we feel much doubt as to the verdict that will be returned, notwithstanding the ungracious insinuation of our learned opponent, that his views can only be resisted "by the interested, by the prejudiced, and by the insufficiently informed."

In consequence of the great extension of our observations on Dr. Copland's article on Pestilential Cholera, we have left ourselves but little space to notice the work of Dr. White on the Plague. But this is not much to be regretted; since, from the very nature of its contents, we must have been utterly precluded from analysing its contents at any length. The *late* plague of Corfu, which it professes to describe, occurred upwards of 30 years ago; and our author, who was superintendant of the infected district of Lefchimo in that island, wrote at that time the present work, without, however, having made up his mind to publish it or not. Why he has deferred its publication so long, is not very obvious. But, as it does not contain even so much as an allusion to what has been done by others since the year 1816, we may be very fairly excused from bringing any portion of its details before our readers. To write in the present day of the Plague without reference to the observations that have been made in various countries during the last quarter of a century, more especially during the last twelve years in Egypt, is like treating of diseases of the chest without mentioning the discoveries of Laennec, or discoursing on animal chemistry with the name and labours of Liebig left out.

Although unable, therefore, to report favourably of Dr. White's treatise ourselves, we willingly give him the benefit of Dr. Copland's recommendation, appended to his article on the Plague. It stands thus:—"This important work, the result of much experience, appeared as this sheet was going to press. It contains numerous additional proofs of the truth of the doctrines for which I have contended." What the nature of these doctrines is, our readers will easily imagine; for, if Epidemic Cholera be so very infectious—that infection from one individual to another has been the chief agent in its dissemination from the shores of the Ganges to those of the St. Lawrence, from the Tropic of Capricorn even to the frozen shores of the Baltic—what must the Plague itself be? It would seem as if the very contemplation of the subject had, in some strange and mysterious way, inoculated our learned author with some of those hot and fiery particles which, according to the doctrines of many of the old physicians, served to inflame the blood and disorder the animal spirits in all malignant fevers. Instead of taking a calm and dispassionate view of the important and much vexed question as to the mode or modes of the transmission of the disease, he treats the whole subject as a special pleader might be supposed would have done, if retained by the employés of quarantine establishments to defend their long-enjoyed and now threatened privileges. Mere declamation and bold assertion are substituted for patient enquiry and sober deduction; while unmeasured, and often most unbecoming, abuse is made to take the place of gentlemanly disputation. The whole article teems with exaggerated statements, with not a few errors and inconsistences intermixed. We have no intention now of canvassing either the data or the conclusions that are adduced. This labour indeed is quite unnecessary, after the ample exposition, which we gave in our number for last October, of the truly admirable report of the French Academy on the Plague. From the very favourable manner in which that exposition has been received by the medical public, we feel quite assured that the views, therein advocated, will be found upon further examination to be both most reasonable and correct. The manner, in which Dr. Copland has treated the French report, is utterly inconsistent, alike with good taste, fair dealing, or sound judgment. All the evidence that militates against his views is most religiously left out, and even the names of some of the most distinguished physicians, who, after so nobly devoting themselves for years to the not unperilous task of examining the real, not the biblical, history of the Plague in the land of its worst malignity, have declared themselves on the opposite side of the question, are not so much as mentioned. It would be easy to adduce passage upon passage from Dr. C.'s narrative, to shew that he has quite forgotten the character of an impartial umpire or judge for that of a zealous and somewhat unscrupulous advocate. The whole of the long note at page 207 is a flagrant instance of the veriest lawyer-like quibbling. What shall we say of an author who, in alluding to the influence of *epidemic foci*—in other words, of localities where (what has been called) an epidemic constitution of the atmosphere prevails—on the diffusion of a pestilential disease, indulges in such language as this?

"Without, however, enquiring into the origin and nature of these 'foyers'—for such enquiry is never thought of by them (the Commissioners of the French Academy), it being quite sufficient to assume their existence—it must be inferred that they are most unaccountable things, seeing that they possess neither length, breadth, nor thickness, nor other material characteristics, and yet produce material effects; that they are neither recognised nor recognisable, and yet they destroy large portions of the human race; that their existence is an hypothesis, a supposition, and yet they produce ruin and devastation; that their hypothetical presence is only for a few weeks or months, and then, after many hundreds of years, never again to return, or then, after short intervals, according to the manner of their reception. How very odd is this occurrence!"

Nay; how very stange is such an unmeaning tirade upon such an occasion! But it is not the French Academicians alone that come in for ridicule and abuse. Every one, that does not hold with our author—who, be it remembered, never saw a case of plague in his life-time—is thus summarily disposed of:—

"I have hesitated to consider these arguments (adduced by those who contend that infection is not the chief agent in the propagation of the disease—*Rev.*) at all; because, when a matter is fully and irrefragably established on facts, any argument which can be brought against it—all special pleadings, however ingenious, argue either the fractious spirit of the objector, or some motive actuating him to prevent a belief in the truth. On this account, therefore, the arguments —or rather the sophistical puerilities, which have been adduced by ignorant, interested, or captious and splenetic persons, hardly deserve a notice, and only when they seem to possess an air of importance—an importance derived only from unwarranted assumptions, confident assertions, and ill-founded pretension; and, in some instances, also from the official or professional position of some of those who have ventured into the field of controversy—and not from any solid array of facts or of inferences logically drawn from facts. But irrespective of the want of every claimant of sound argument, a very large proportion of the Anti-infectionists betray, as shewn above, an utter ignorance of the distempers respecting the nature of which they speak with confidence, and even with disgusting pretension—and not only of these distempers, but even of others, either allied or analogous to them, of which they have incidentally taken notice. This assertion may be conceived by some, who have not had opportunities of judging for themselves in the matter, as severe or ill-founded; but it could be very easily proved, if it deserved the space which would be wasted in proving it."

The only comment we shall make upon this passage is to quote the opinion of a gentleman who has, within the last few years, had a good deal of experience in the disease of which he treats, but to whom Dr. Copland makes no reference; we mean Dr. Robertson, deputy-inspector of hospitals, who served with our troops lately engaged in Syria. In 1841, he thus expressed himself in his official report to the Government:—

"In reference to the contagious or non-contagious* nature of this at times frightful disease, I beg to state that the result of all my experience leads me to believe that the disease originates in local causes, and that it is endemic in Syria and Egypt; that it is not of a highly contagious nature; and that, if ever so at all, some other concurrent circumstances are necessary to render it so. Extreme and exclusive opinions on the doctrine of contagion are hardly warranted by the present state of our knowledge. My own firm conviction is, that the Plague cannot be communicated from one person to another in a pure atmosphere, even by contact; but I am not prepared to assert that, if plague-patients are crowded together in confined and ill-ventilated apartments, infection will not be produced, just as happens in Typhus fever."

We are glad to observe that our Government evinces every disposition to introduce a relaxation of the existing Quarantine Regulations. The recent removal of all quarantine upon vessels (with a clean Bill of Health) arriving from any port in the Turkish dominions, is but an earnest of still greater and more important alterations.

We have now examined the history of two of the Pestilences described by Dr. Copland; in our next we propose to bring the third, viz. the Yellow Fever, under the notice of our readers.

---

* These words are here used in the general sense of "transmissible" and "non-transmissible."—*Rev.*

